# A genetically distinct hybrid zone occurs for two globally invasive mosquito fish species with striking phenotypic resemblance

**DOI:** 10.1002/ece3.2562

**Published:** 2016-10-24

**Authors:** Rebecca J. Wilk, Lisa Horth

**Affiliations:** ^1^ Department of Biological SciencesOld Dominion UniversityNorfolkVAUSA

**Keywords:** climate change, fish, genetic, hybridization, hybrids, microsatellite

## Abstract

Hybrid zones allow for the investigation of incipient speciation and related evolutionary processes of selection, gene flow, and migration. Interspecific dynamics, like competition, can impact the size, shape, and directional movement of species in hybrid zones. Hybrid zones contribute to a paradox for the biological species concept because interbreeding between species occurs while parental forms remain distinct. A long‐standing zone of intergradation or introgression exists for eastern and western mosquito fish (*Gambusia holbrooki and G. affinis*) around Mobile Bay, AL. The region has been studied episodically, over decades, making it perfect for addressing temporal dynamics and for providing a deeper understanding of the genetics of these periodically reclassified fishes (as species or subspecies). We used six microsatellite markers to assess the current population structure and gene flow patterns across 19 populations of mosquito fish and then compared our results with historical data. Genetic evidence demonstrates that the current hybrid zone is located in a similar geographic region as the historical one, even after three decades. Hybrid fish, however, demonstrate relatively low heterozygosity and are genetically distinct from western and eastern mosquito fish populations. Fin ray counts, sometimes used to distinguish the two species from one another, demonstrate more eastern (*G. holbrooki)* phenotype fish within the molecular genetic hybrid zone today. Mosquito fish are globally invasive, often found on the leading edge of flooded waters that they colonize, so the impact of hurricanes in the wake of climate change was also evaluated. An increase in the frequency and intensity of hurricanes in the hybrid region has occurred, and this point warrants further attention since hurricanes are known to move these aggressive, invasive species into novel territory. This work contributes to our classical understanding of hybrid zone temporal dynamics, refines our understanding of mosquito fish genetics in their native range, evaluates important genotype–phenotype relationships, and identifies a potential new impact of climate change.

## 1 Introduction

Hybrid zones pose a clear paradox to the biological species concept (Mayr, 1942, 1963) since two species can interbreed in them while parental forms remain distinct (Barton & Hewitt, 1985). Recognizing this paradox has resulted in a quest for greater understanding of hybrid zone dynamics, which continue to be explored today. In 1977, Endler (1977) constructed a model addressing how the geographic differentiation of species could actually evolve across continuous populations, and how steep clines could arise when connected populations that were previously isolated became re‐isolated. Later, Barton and Hewitt (1985) demonstrated that a “dominant” species, with higher adaptive fitness to a given niche, could drive asymmetric introgression, and production of hybrids with more “dominant” species’ alleles (Barton & Hewitt, 1985). Phenotypic evidence of this occurs when hybrids physically resemble the “dominant” species (Buggs, 2007). Hybridization can facilitate adaptive differences between species, and also cause interspecific competition, as a result of range overlap (Hoffmann & Sgrò, 2011).

Recent attention has been drawn to introgressive hybridization and the associated loss in biodiversity resulting from anthropogenic translocations of invasive species (Allendorf, Leary, Spruell, & Wenburg, 2001; Seehausen, 2006). For example, when the Signal crayfish (*Pascifacticus lenisculus*) invaded Enos Lake (Vancouver Island, CA), standing macrophytes and water clarity were disrupted. A mating barrier breakdown then occurred between the sympatric benthic and lentic three‐spined sticklebacks (*Gasterosteus aculeatus*). A substantial uptick in hybrid frequency raised concern about native species loss and the formation of a new hybrid swarm (Kraak, Mundwiler, & Hart, 2001).

The southeastern United States harbors one of the world's most diverse freshwater fish assemblages, with the Mobile Bay, AL basin ranked third in species richness (*n* = 157) for North America. The basin contains 40 endemic species (Swift, Gilbert, Bortone, Burgess, & Yerger, 1986), and the region is recognized as a zone of high intergradation. Geological changes, glaciation, and concomitant sea level fluctuation have caused dramatic alterations in habitat availability in the region, with repeated periods of habitat isolation and connectedness for freshwater fishes.

**Figure 1:**
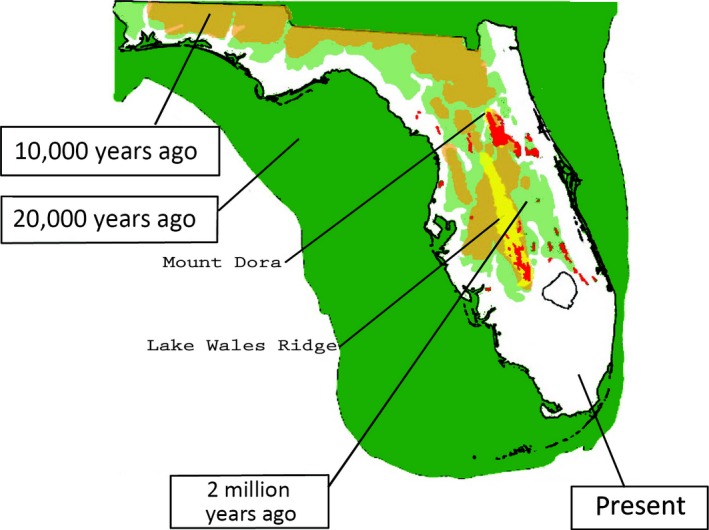
Historical biogeography of Florida. The red patches represent relict scrub habitat from the Pliocene or earlier. The light green patchy distribution represents land above sea level 2 M ybp and the yellow patch represents the Lake Wales Ridge, a relatively high ridge probably formed during the Pleistocene. The large green tract represents the entire land mass 20 K ybp. The golden color represents the land mass 10 K ybp. The white area represents FL today. Reprinted from Germain‐Aubrey et al. (2014)

During the Oligocene Epoch, the Coastal Plain emerged for the first time in the region south of the AL uplands (Swift et al., 1986). However, this was followed by marine and brackish water re‐inundating the area during the Miocene. Even in the early Pliocene, nearly all of the southeastern shoreline remained underwater, including coastal LA, east to the FL panhandle, and south through the Everglades (Figure 1; Germain‐Aubrey et al., 2014; Webb, 1990). A few small island habitats existed in FL (e.g., Tifton–Tallahassee uplands, Western Highlands of northwest FL, and Lake Wales Ridge, Hendry & Sproul, 1966) and rising terrain occurred at the northern end of Mobile Bay, AL, extending northwesterly (including the Hatchetigbee anticline, Wiggins uplift, Jackson Dome, Monroe uplift, faults and salt domes; Murray, 1961 and Swift et al., 1986).

Later, during the Pleistocene, glaciers and interglacial stages led to episodic freshwater availability in the coastal and peninsular regions (Bermingham & Avise, 1986). Habitat loss forced freshwater fishes to migrate northerly, westerly, or face extinction (Swift et al., 1986). Peninsular FL fish populations were sometimes relegated to small habitat fragments, like the Ocala Highlands (Bermingham & Avise, 1986). As sea level fell, southerly and westerly migrations occurred, coincident with glacial movement. By the late Pleistocene, the coastline surrounding the Gulf of Mexico had expanded seaward (Figure 1). Since then, the Gulf coast has again receded, so fish extant today must have migrated inland to survive. The sandy Coastal Plain in southern AL is sharply delineated from the northeastern rocky Piedmont by a distinct Fall Line, and streams in eastern MS and western AL still reflect patterns related to the old Pliocene uplift (Burnett & Schumm, 1983).

The Mobile Bay area is also considered a “boundary” region for a fair number of clades of freshwater fish species, subspecies, and disjunct populations, indicative of a large‐scale pattern in the area (Wiley & Mayden, 1985). In this work, we present genetic evidence that a long‐standing hybrid zone remains occupied by mosquito fish (western mosquito fish, *Gambusia affinis*, and eastern mosquito fish, *G. holbrooki*) in the Mobile Bay, AL region. Both species are highly aggressive, globally invasive freshwater fishes, native to the southeastern United States. *G. affinis* has a native range west of the hybrid zone into parts of TX and NM, and north through several mid‐western states. *G*. *holbrooki* has been considered native eastward of Mobile Bay, south throughout peninsular FL, and north up the eastern seaboard to NJ. These fishes have been introduced to every continent except Antarctica, and to over 50 countries (García‐Berthou et al., 2005), with little regard to their invasive nature, or their impact on native and endangered species, which they sometimes annihilate (Pyke, 2008). Mosquito fish reproduce rapidly and profusely. They are aggressive in both mating and predatory behavior (Alcaraz, Bisazza, & Garcia‐Berthou, 2008; Horth, 2003; Pyke, 2008). They can distribute widely, tolerating many aquatic environments, including brackish water (Stearns & Sage, 1980).

The taxonomy of mosquito fish has been revised repeatedly over time, beginning with *Gambusia affinis* (synonym *Gambusia affinis affinis*) (Baird & Girard, 1853) and *Gambusia holbrooki* (Girard, 1859). In 1939, *G. holbrooki* and *G. affinis* were reclassified as a single species (D'Ancona, 1939). A few decades later, Rivas (1963) suggested redesignating them subspecies, an historical classification also used by Rosen and Bailey (1963) based upon several phenotypic traits, including fin ray counts. Over the last century, the Mobile Bay, AL region has been crucial to understanding genetic and phenotypic differentiation in these fishes (Angus & Howell, 1996; Black & Howell, 1979; Hubbs, 1955; Wooten, Scribner, & Smith, 1988). In 1988, Wooten et al. (1988) presented genetic evidence to again suggest a two species designation. They found species’ ranges akin to those proposed 25 years earlier by Rosen and Bailey (1963) and an intergradation zone in the general region that Hubbs (1955) found noteworthy 33 years earlier.

Wooten et al. (1988) identified low gene flow, abrupt allele frequency changes, and high differentiation. Nearly a decade later, Angus and Howell (1996) used fin counts, sampled extensively within the zone, and found that 85% of sites (33 of 39) in the historical hybrid region were dominated by *G. holbrooki* with far fewer hybrid fin count fish. Introgression has been documented quite specifically for *Gambusia* phenotypes in the northern portion of the Mississippi River drainage basin. The upper Conasauga River system, which empties into Mobile Bay, was sampled prior to 1984 when all fish collected were considered *G. affinis* (Angus & Howell, 1996; Rosen & Bailey, 1963; Walters 1997). In 1984, the first *G. holbrooki* was collected from the river system based upon gonopodial traits and fin ray counts (Walters & Freeman, 2000) and a little over a decade later (1996–1998), seven locations were sampled extensively: Three contained traits that matched *G. holbrooki*, two contained both species plus hybrids, and two more sites contained both species but few (<10%) hybrids. All seven locations were southern creeks, meaning fish were migrating northward from the region closer to the Gulf of Mexico (Walters & Freeman, 2000) and *G. holbrooki* were thought to have been recent colonizers of the river system, based on the low number of hybrids detected.

Past empirical work demonstrated that hybridization occurred rapidly between the species and that *G. affinis* nuclear and cytoplasmic alleles decreased within just a few generations (Scribner & Avise, 1994). Evidence was found that female *G. holbrooki* prefer conspecific over heterospecific males and mate with conspecific individuals more often (84% of matings, Scribner, 1992). As *G. affinis* females mated randomly across species, a greater loss of *G. affinis* alleles might be anticipated in the hybrid zone. *G. affinis* have also been shown to prefer larger males, which could be relevant in the hybrid zone since *G. holbrooki* have been shown to be larger (Deaton, 2007).

*Gambusia holbrooki* have an XX–XY sex determination system, with heterogametic males; *G. affinis* have a WZ–ZZ system, with heterogametic females (Black & Howell, 1979), resulting in some mating incompatibility. In past work, when *G. holbrooki* females (XX) hybridized with *G. affinis* males (ZZ), the F_1_ generation produced was 100% male (XZ), but when *G. affinis* females (WZ) hybridized with *G. holbrooki* males (XY), the F_1_ generation was, on average, 50–75% male (ZY, XZ, possibly WY) and 25–50% female (WX, possibly WY) (Lucas & Southgate, 2012; Schultheis, Bohne, Schartl, Volff, & Galiana‐Arnoux, 2009) with some offspring, severely deformed which resulted in death (Black & Howell, 1979). Since the latter cross produced less viable offspring, relatively more hybrids could inherit *G. holbrooki* maternal genes and mtDNA in a natural hybrid zone.

Assessing genetic structure in hybrid zones temporally over decades is rare. In this work, mosquito fish population genetic structure and phenotypic (fin ray counts) structure were evaluated and compared to historical data (Angus & Howell, 1996; Scribner & Avise, 1993; Wooten et al., 1988). We used six polymorphic microsatellite loci to evaluate genetic patterns. Gene flow and population structure were evaluated. Given the highly destructive nature of these species, when invasive, to other native species (Goodsell & Kats, 1999), a climate change variable was also assessed: Hurricane patterns were evaluated for the region since severe weather has a history of physically moving mosquito fish inland in the Gulf of Mexico region, potentially impacting other sensitive species.

Genetically, we identified three distinct clusters (western, hybrid, and eastern) of *Gambusia* that presently exist along the Gulf of Mexico. The genetic hybrid zone occurs in the same general region as intergradation was previously identified. However, *G. holbrooki* phenotypes have migrated westerly and hybrid genotype fish often express them. Additionally, hurricane patterns suggest that the potential for movement of these fishes increases with climate change.

## 2 Materials and Methods

### 2.1 *Gambusia* collection and molecular genetic analysis

**Figure 2:**
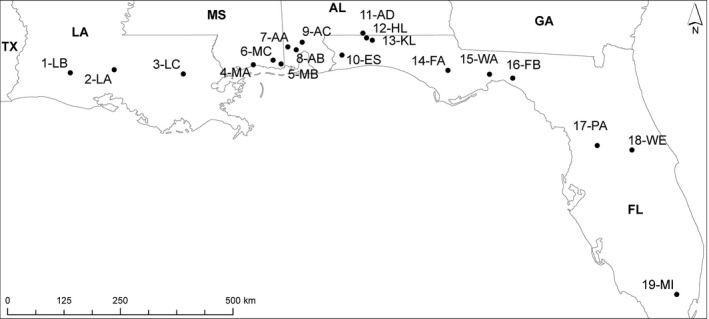
Mosquito fish collections sites [STATE of collection (site acronym: latitude, longitude)]: LA (1‐LB: 30.248, −92.665; 2‐LA: 30.311, −91.785; 3‐LC: 30.225, −90.412), MS (4‐MA: 30.408, −89.014; 5‐MB: 30‐.424, −88.461; 6‐MC: 30.618, −88.621), AL (7‐AA: 30.763, −88.327; 8‐AB: 30.707, −88.159; 9‐AC: 30.855, −88.040; 11‐AD: 31.032, −86.829), FL (10‐ES: 30.599, −87.247; 12‐HL: 30.943, −86.755; 13‐KL: 30.896, −86.642; 14‐FA: 30.295, −85.132; 15‐WA: 30.219, −84.302; 16‐FB: 30.141, −83.839; 17‐PA: 28.800, −82.154; 18‐WE: 28.712, −81.460; 19‐MI: 25.838, −80.567)

About 30 individual fish were collected (from 2011 to 2014) for molecular analysis from each of 19 populations in the native range (Figure 2), including nine FL, four AL, three MS, and three LA populations. All regions were previously reported upon for fin ray counts (Angus & Howell, 1996) so in this work, a subset of populations around the hybrid region were analyzed for current fin ray counts. Fish were euthanized with MS‐222 on site and transported back to Old Dominion University (Norfolk, VA) on ice, then stored at −80°C.

Total genomic DNA was extracted from tail (postabdomen to caudal fin) muscle tissue using the Qiagen DNeasy Blood and Tissue Kit (Valencia, CA, USA). Samples were assessed for quality and quantity using a Thermo Scientific NanoDrop 1000 spectrophotometer (Wilmington, DE, USA). Polymerase chain reaction (PCR) was run for six microsatellite loci using fluorescently labeled primers for Gafμ 1, Gafμ 2, Gafμ 3, Gafμ 4, Gafμ 6, and Gafμ 7 (Spencer et al., 1999). Two nonoverlapping primers sets were multiplexed per reaction (Appendix S1). Gafμ 1 and Gafμ 4 were duplexed, as well as Gafμ 2 and Gafμ 6, and Gafμ 3 and Gafμ 7. 25 μl reactions were run (5 μl DNA at ~20 ng/μl, 5 μl RNase free water, 0.625 μl each primer (0.1 μmol/L concentration), 12.5 μl of mastermix from Qiagen (Type‐It Microsatellite Kit, Valencia, CA, USA) for 5:00 min at 95°C, 35 cycles for 0:30 s at 95°C, 1:30 min at 64°C, 0:30 s at 72°C, 10:00 min at 68°C.

Polymerase chain reaction products were processed on an ABI 3130xl (Applied Biosystems, Grand Island, NY, USA) genetic analyzer. Forward primers were fluorescently labeled with FAM or HEX dye. Peak calls were made manually using GeneMapper v. 3.7 and Peak Scanner 1.0 (Applied Biosystems) and rounded to the nearest whole number. Bins were created and subsequently compared to previously reported microsatellite reads for *G. affinis* (Spencer, Neigel, & Leberg, 2000). Approximately 10% of samples were run a second time for read verification.

MICRO‐CHECKER v. 2.2.3 was used to assess the accuracy of allele scoring by checking for stutters, large allele dropouts, and null alleles (Van Oosterhout, Hutchinson, Willis, & Shipley, 2004). Stutters may occur when a deficiency of heterozygote genotypes with alleles of one repeat unit difference exists. Null alleles demonstrate an excess of homozygotes over most allele size classes, but are actually unlikely to change assignment results (Carlsson, 2008).

GENEPOP 4.2 (Raymond & Rousset, 1995; Rousset, 2008) and ARLEQUIN 3.5 (Excoffier & Lischer, 2010) were used to assess Hardy–Weinberg equilibrium (HWE), heterozygosity, allelic frequencies, and private alleles. FSTAT 2.9.3 (Goudet, 2001) was used to calculate allelic richness and inbreeding coefficients.

Microsatellite data files were converted to necessary formats using PGD SPIDER 2.0.8.2 (Lischer & Excoffier, 2012). STRUCTURE 2.3.4 was used with permutations set to 1,000 to assess population structure, the hybrid zone, migrating individuals, and allele frequencies (Pritchard, Stephens, & Donnelly, 2000). STRUCTURE HARVESTER 0.6.94 was used to determine the appropriate *K*‐value (or number of clusters) (Earl & von Holdt, 2012) based upon three sets of stringencies: (1) all samples and no population designation, (2) all samples and assigned populations, and (3) all populations with designations, but excluding data for locus Gafμ 6 (which was out of HWE for several populations and demonstrated potential stuttering). Each K scenario (up to 15 clusters) was simulated three times in STRUCTURE and averaged in STRUCTURE HARVESTER.

Mean log probability y of data (ln P(D)) and change in *K* (delta *K*) values were produced to determine the correct number of clusters (*K*) for use with STRUCTURE. All runs were conducted with a burn‐in period of 100,000 (and 1,000,000) iterations. Individuals adhering to their respective assigned STRUCTURE cluster by >80% agreement of associated genotype were considered to be in their assigned cluster (Andrews, Norton, Fernandez‐Silva, Portner, & Goetze, 2014; Gauthier et al., 2013).

ARLEQUIN 3.5 (Excoffier & Lischer, 2010) was then used for analysis of molecular variance (AMOVA) to detect variation within and between populations, and to calculate pairwise *F*_ST_ values. Mantel tests were run in R using ade4 and vegan packages to detect spatial autocorrelation between matrices (Dray & Dufour, 2007; Oksanen et al., 2015). In the Mantel test, genetic distances were estimated between population pairs based on *F*_ST_ values and the shortest distance between geographic locations (GPS coordinates). A partial Mantel test was run with *F*_ST_ values, STRUCTURE 2.3.4 clusters, and GPS coordinates to test whether isolation by distance results could have been falsely positive due to clustering (Meirmans, 2012). The tests were based on Pearson's product–moment correlation with 999 permutations.

### 2.2 Phenotype analysis

**Figure 3:**
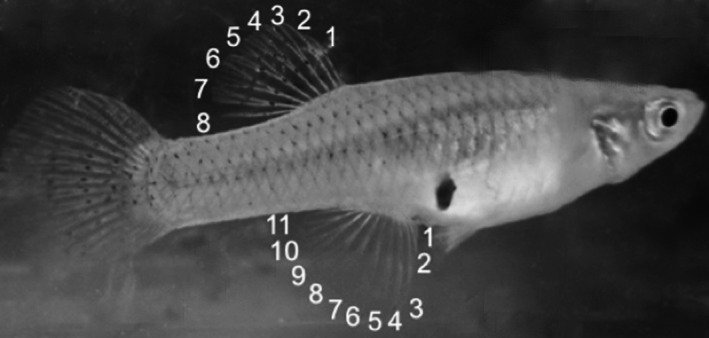
*Gambusia holbrooki* female fin rays contain eight dorsal and eleven anal rays

Each fish was placed under a dissecting microscope and two people independently counted fin rays. Upon comparison of counts, few discrepancies were identified and resolved. Rays were individually separated and counted as though they were not branched. Using this method, *G. holbrooki* typically has eight dorsal fin rays and 11 anal rays and *G. affinis* has seven and 10, respectively (Rivas, 1963; Walters & Freeman, 2000). Approximately 40 fish from each of 10 populations across the hybrid region were used. Only females can be hybrid phenotypes since the anal fin of males transforms into a gonopodium (intromittent organ) at maturation. Female dorsal and anal fin rays were counted (Figure 3) to evaluate phenotypes. Male dorsal fins were also counted and these were used to assess frequencies of male *G. affinis* and *G. holbrooki* phenotypes. The frequency of female *G. affinis*, hybrids, and *G. holbrooki* phenotypes were plotted by location using ArcMap 10.2.2 (ESRI 2014).

To compare genotype and phenotype, each individual was assigned to a category (species or hybrid) based upon fin ray count and then compared to the genetic cluster assignment (eastern, western, or hybrid), considering also private and semiprivate alleles identified for each species. Due to the presence of potential null alleles in some of these populations for two loci (Gafμ 4 and Gafμ 6), every individual's genotype was assessed for four loci (Gafμ 1, Gafμ 2, Gafμ 3, and Gafμ 7), allowing for designation as pure *G. affinis*,* G. holbrooki*, or hybrid for the comparison to fin ray count. All (other) molecular analyses included all markers (discussed elsewhere).

### 2.3 Comparison of present patterns to historical patterns

The results from two historical genetic studies evaluating intergradation and hybridization around the Gulf of Mexico were compared to our genetic results. Wooten et al. (1988) comprehensively evaluated allozymes for mosquito fish in the southeast and graphically presented two variable loci that have been reproduced on our map of the region. Scribner and Avise (1993) analyzed five allozyme loci along with mtDNA and combined these results to categorize populations based on the frequency of *G. affinis* alleles. The Scribner and Avise (1993) mtDNA data were also included on our map, along with our own molecular results, for comparative purposes. Both past studies were highly valuable for determining population‐level frequencies of *G. affinis* and *G. holbrooki* alleles.

We constructed a second, similar figure where we layered our fin ray count data atop a geographic map with Angus and Howell's (1996) historical fin ray counts, for temporal comparison. Both images were completed using ArcGIS (Environmental Systems Resource Institute (ESRI), 2014).

### 2.4 Hurricanes impacting the hybrid region

Major weather patterns are well known to impact population connectedness and gene flow. In the Gulf of Mexico, forceful hurricanes occur frequently and their paths include the hybrid zone. The frequency and intensity of historical hurricanes (1900–2013) was plotted for the Western Atlantic Ocean and Gulf of Mexico (NOAA 2015) and evaluated with linear regression (SPSS v 22.0, IBM Corp. 2013). The distribution, direction, and intensity of recent hurricanes affecting the hybrid region (1985–2013) were determined using NOAA's hurricane mapper tool. The diameter and path of hurricanes with the greatest impact in the region of the hybrid zone were plotted using GIS for evaluation (image not shown).

## 3 Results

### 3.1 Molecular genetic analysis

Power analyses of sample size prior to this assessment indicated that 30 mosquito fish per population were sufficient to identify the important genetic relationships within and among populations (Table 1, Appendix S2). MICRO‐CHECKER results showed no signs of large allele dropout for any locus, and potential stuttering was only exhibited for Gafμ 6.Two populations had potential stuttering and a possible null allele for Gafμ 6. Four populations had only a possible null allele for Gafμ 4, one population for Gafμ 3, one for Gafμ 7, and six additional populations for Gafμ 6. ARLEQUIN results where P values were Bonferroni‐corrected for multiple comparisons (*K* = 19) showed that four populations were out of HWE for Gafμ 6, and two populations were out for Gafμ 4. Due to potential stuttering and disequilibrium for four populations for Gafμ 6, all further genetic tests were conducted twice: (1) all populations and all loci, and (2) all populations and all loci, excluding Gafμ 6. Given the similarity of results, we report results for all populations and all loci since statistical significance did not vary between (1) and (2).

**Table 1:** Summary of genetic diversity data from 19 populations in the southeastern United States, averaged over six loci listed from west to east

	*H*_o_	*H*_e_	AR	*F*_IS_	G‐W
1LB	0.813	0.822	7.508	0.012	0.240
2LA	0.667	0.682	8.305	0.024	0.349
3LC	0.632	0.691	8.421	0.087	0.293
4MA	0.774	0.761	8.076	−0.018	0.245
5MB	0.489	0.497	6.918	0.017	0.267
6MC	0.538	0.537	6.840	−0.002	0.210
7AA	0.557	0.595	6.318	0.064	0.283
8AB	0.495	0.537	6.281	0.080	0.212
9AC	0.629	0.680	7.887	0.076	0.279
10ES	0.783	0.813	6.908	0.037	0.212
11AD	0.786	0.766	6.833	−0.026	0.203
12HL	0.756	0.827	7.952	0.088	0.318
13KL	0.745	0.782	7.166	0.048	0.302
14FA	0.606	0.682	7.746	0.114	0.300
15WA	0.767	0.811	9.345	0.055	0.323
16FB	0.756	0.805	9.192	0.062	0.334
17PA	0.722	0.830	9.136	0.132	0.366
18WE	0.722	0.791	9.370	0.088	0.325
19MI	0.786	0.881	10.381	0.110	0.323

*H*_o_, observed heterozygosity; *H*_e_, expected heterozygosity; AR, allelic richness adjusted for smallest population; *F*_IS_, inbreeding coefficient; G‐W, Garza–Williamson Index.

**Figure 4:**
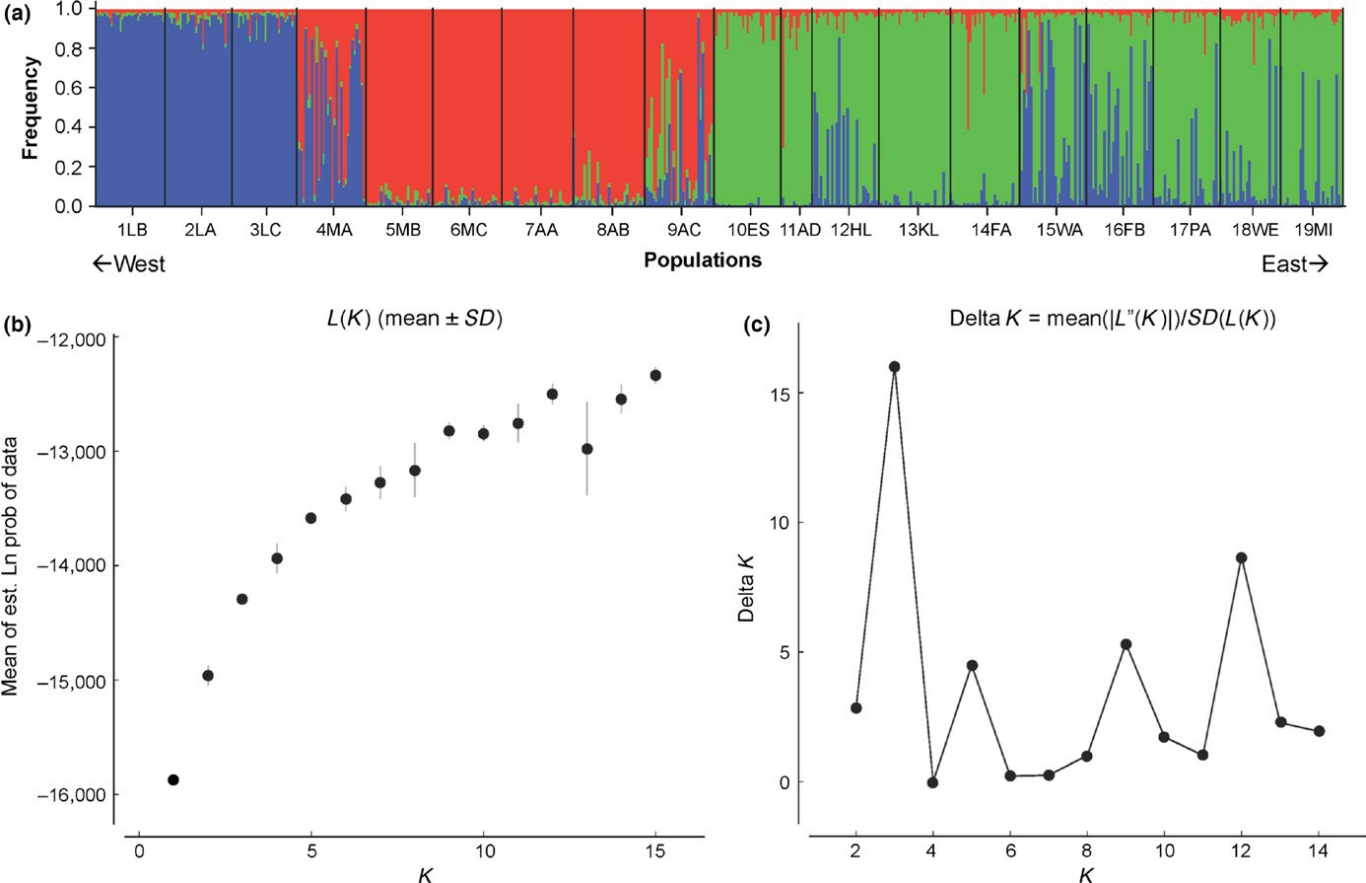
(a) STRUCTURE plot for distinct clusters (*K* = 3) from west to east (b) *K*‐value determined by Ln P(D) (c) Appropriate *K*‐value determined by delta *K*

Microsatellite data (Appendix S3) in STRUCTURE demonstrated three genetic clusters (Figure 4a). The western genetic cluster consisted of the three most westerly geographic populations (LA; populations 1–3 in blue, Figure 4a), the hybrid cluster consisted of the six geographically central populations (five MS, one western AL; populations 4–9 in red, Figure 4a), and the eastern cluster consisted of ten geographically eastern populations (one eastern AL, nine FL; populations 10–19 in green, Figure 4a). A K‐value of three was used in this CLUSTER analysis, since the LnP(D) for three had the highest likelihood (Figure 4b) and the delta *K* for 3 showed the greatest change (Figure 4c).

For the western cluster, no individuals deviated genetically from the cluster. For the hybrid cluster, 41 individuals (23%) deviated genetically, with the vast majority (90%) of these (37 of 41) individuals being from the two most peripheral populations of the hybrid cluster (4‐MA and 9‐AC). For the eastern cluster, about a quarter (24%) of the individuals (67 of 280) deviated genetically, with most mismatches actually corresponding to the western cluster. Results from the partial Mantel test (three matrices) were highly significant (*r* = .6677, *p* = .001); those from the regular Mantel test (two matrices) were not (*r* = .05586, *p* = .317).

Observed heterozygosity (*H*_0_) differed significantly among the three genetic clusters identified (western *H*_0_ = 0.659, hybrid *H*_0_ = 0.580, eastern *H*_o_ = 0.740; *p* = .034), although other population genetic variables did not, including allelic richness (AR) (western AR = 8.0; hybrid AR = 7.0, eastern AR = 8.4; *p* = .184), average inbreeding (*F*_IS_, coefficient, based upon Weir and Cockerham (1984); western *F*_IS_ = 0.40, hybrid *F*_IS_ = 0.36, eastern *F*_IS_ = 0.76; *p* = .284), and average subpopulation structure (*F*_ST_; western *F*_ST_ = 0.022, hybrid *F*_ST_ = 0.072, eastern *F*_ST_ = 0.086). Comparison of populations within and among clusters demonstrated that the highest allelic diversity was present in eastern mosquito fish.

Interesting patterns arise when assessing individual hybrid cluster populations. The hybrid population farthest west (4‐MA) was a clear mix of *G. affinis* and hybrid alleles (Figure 4a). However, the next population eastward (5‐MB) had the lowest heterozygosity of all populations (Table 1) and was overwhelmingly dominated by hybrid alleles, just like the next three populations eastward (6‐MC, 7‐AA, 8‐AB; Figure 4a). Population 8‐AB had the lowest allelic richness of all populations, but the next most easterly population in the hybrid zone (9AC) had the highest heterozygosity observed (Table 1) and a clear mix of *G. holbrooki* and hybrid alleles, suggesting some important differences in the peripheral region of the hybrid zone relative to the more central region.

The Garza–Williamson (G‐W) index represents the ratio between total number of alleles and allelic range. Particularly low G‐W values may reflect reduced effective population sizes (Ne), or bottlenecks. The G‐W index was lowest for the hybrid cluster, intermediate for the western cluster, and highest for the eastern cluster populations (Table 1).

Differences in numbers of private alleles can reflect ancestral alleles, or allelic expansion frequency differences. The western cluster had the fewest (*n* = 2) private alleles, the hybrid cluster an intermediate number (*n* = 11), and the eastern cluster the most (*n* = 21). This same rank held true for the average number of private alleles per population (western = 0.67, hybrid = 1.83, eastern = 2.1, Table 1). The relatively high number of *G. holbrooki* populations sampled could also increase values for the eastern cluster.

AMOVA results (Table 2) from ARLEQUIN demonstrated the highest genetic variation within populations (83.63%) and the least genetic variation among populations within clusters (6.90%). The three largest pairwise *F*_ST_ values, which reflect the greatest population‐level differentiation, occurred between the hybrid cluster population that is the second most westerly (5‐MB) in the hybrid zone, and each of the other western cluster populations (1‐LB, 2‐LA and 3‐LC, *F*_ST_ = 0.251, 0.248 and 0.258 respectively, Table 3). Interestingly, the one population further west within the hybrid cluster (4‐MA) had greater similarity to the western cluster populations (*F*_ST_ = 0.085, 0.100 and 0.092, respectively, Table 3), which is also clearly visible in the STRUCTURE output (Figure 4a).

**Table 2:** Analysis of molecular variance (AMOVA) results for six microsatellite loci in mosquito fish populations in the southeastern United States

Source of variation	*df*	SS	VC	%Var	*p* Value
Among groups	2	191.043	0.24285	9.47	<.00001
Among populations	16	200.056	0.17689	6.9	<.00001
Within populations	1099	2357.191	2.14485	83.63	<.00001

*df*, degrees of freedom; SS, sum of squares; VC, variance components; %Var, percentage of variation explained by source of variation; *p* value, statistical significance of AMOVA test result, where *p* ≤ .05 is significant.

**Table 3:** Pairwise *F*_ST_ values for 19 collections of mosquito fish, from west to east, in the southeastern United States. All values are significant (*p* < .001) except the value with an asterisk (comparing two western populations, 2‐LA and 3‐LC). *F*_ST_ values that indicate the highest differentiation are bold (where western populations are compared to hybrid population 5‐MB) and those that indicate the lowest differentiation are underlined (comparing hybrid populations within the hybrid zone, and comparing some eastern populations within FL, outside the hybrid zone)

	West			Hybrid						East								
	1 LB	2 LA	3 LC	4 MA	5 MB	6 MC	7 AA	8 AB	9 AC	10 ES	11 AD	12 HL	13 KL	14 FA	15 WA	16 FB	17 PA	18 WE
West																		
1 LB																		
2 LA	0.028																	
3 LC	0.037	0.002*																
Hybrid																		
4 MA	0.085	0.100	0.092															
5 MB	**0.251**	**0.248**	**0.258**	0.120														
6 MC	0.227	0.234	0.237	0.092	0.022													
7 AA	0.211	0.209	0.214	0.099	0.057	0.047												
8 AB	0.237	0.231	0.238	0.119	0.070	0.073	0.059											
9 AC	0.130	0.127	0.131	0.047	0.082	0.053	0.059	0.070										
East																		
10 ES	0.191	0.187	0.194	0.142	0.237	0.222	0.190	0.214	0.147									
11 AD	0.215	0.210	0.219	0.150	0.220	0.213	0.170	0.200	0.143	0.027								
12 HL	0.117	0.121	0.116	0.084	0.194	0.183	0.145	0.183	0.110	0.099	0.103							
13 KL	0.189	0.190	0.190	0.131	0.238	0.232	0.206	0.205	0.159	0.093	0.111	0.080						
14 FA	0.181	0.188	0.184	0.146	0.219	0.201	0.156	0.211	0.129	0.162	0.163	0.124	0.181					
15 WA	0.079	0.084	0.081	0.067	0.212	0.183	0.159	0.194	0.090	0.108	0.135	0.055	0.106	0.118				
16 FB	0.095	0.099	0.093	0.075	0.205	0.182	0.154	0.190	0.094	0.121	0.135	0.058	0.121	0.113	0.020			
17 PA	0.121	0.119	0.116	0.098	0.228	0.209	0.180	0.199	0.119	0.099	0.138	0.059	0.068	0.128	0.042	0.048		
18 WE	0.098	0.105	0.106	0.066	0.197	0.172	0.151	0.177	0.072	0.108	0.131	0.067	0.089	0.124	0.032	0.041	0.042	
19 MI	0.130	0.123	0.114	0.092	0.215	0.191	0.163	0.193	0.104	0.069	0.091	0.036	0.071	0.114	0.043	0.043	0.021	0.050

Applying the same pairwise comparisons to the eastern edge of the hybrid cluster produces a similar result: Comparing the second most easterly population in the hybrid cluster (8‐AB) to the three nearby eastern cluster populations (10‐ES, 11‐AD, 12‐HL) produces greater differentiation (*F*_ST_ = 0.214, 0.200, and 0.183, Table 3) than comparing the most easterly hybrid cluster population to these same three eastern populations (*F*_ST_ = 0.147, 0.143, 0.110, Table 3). Thus, population structure is quite different for the peripheral populations in the hybrid zone, relative to the more central populations. Virtually no genetic differentiation existed among the three western (LA) populations, or across all of the eastern peninsular FL populations (Table 3), so the historically “pure species” populations demonstrate relatively low genetic differentiation. All pairwise *F*_ST_ values were significant except for one, which was a comparison of two LA populations (2‐LA and 3‐LC) that were not different from one another (Table 3).

### 3.2 Phenotype analysis

**Figure 5:**
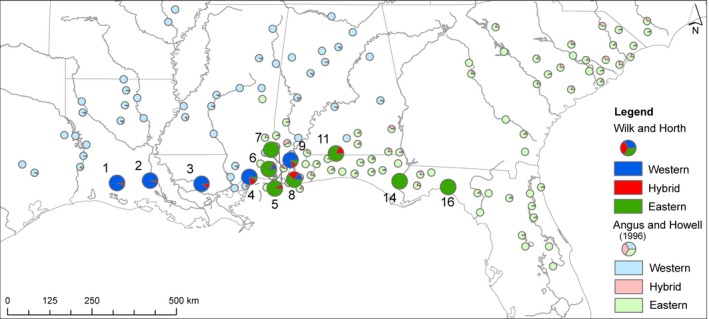
Fin ray counts (Wilk and Horth) and historical fin ray count patterns (Angus & Howell, 1996) for mosquito fish. Bright blue, red, and green pies represent Wilk and Horth's current fin ray data (western, hybrid, and eastern, respectively). Pale blue, red, and green pies represent Angus and Howell's (1996) counts (western, hybrid, and eastern, respectively)

Spanning most hybrid cluster populations (*n* = 4 of 6 hybrid populations), fish with *G. holbrooki* fin ray counts vastly outnumbered fish with western or hybrid fin ray counts (Table 4, Figure 5). The remaining two populations in the hybrid cluster (the populations furthest west and east in this cluster, 4‐MA and 9‐AC, Figure 4a) had predominantly *G. affinis* fin rays counts (Table 4, Figure 5). All eastern cluster FL populations were comprised completely of *G. holbrooki* counts (Table 4, Figure 5). The one eastern cluster population found in AL (11‐AD) was comprised entirely of *G. holbrooki* counts (Table 4, Figure 5), except for two fish (with hybrid fin counts): Of these two hybrid fin count fish, one possessed only eastern alleles and another possessed semiprivate alleles from both eastern and western clusters.

**Table 4:** Raw phenotypic data for fin ray counts, including both sexes

Population	Eastern mosquito fish, *Gambusia holbrooki*	Hybrid	Western mosquito fish, *Gambusia affinis*	Total sample size	Structure cluster
1‐LB	0	1	31	32	Western
2‐LA	0	1	28	29	Western
3‐LC	0	3	27	30	Western
4‐MA	1	5	20	26	Hybrid
5‐MB	24	4	1	29	Hybrid
6‐MC	21	1	2	24	Hybrid
7‐AA	29	0	1	30	Hybrid
8‐AB	20	4	5	29	Hybrid
9‐AC	1	0	31	32	Hybrid
11‐AD	12	2	0	14	Eastern
14‐FA	10	0	0	10	Eastern
16‐FB	8	0	0	8	Eastern

Overall, the vast majority of female fish in the hybrid zone (91%, *n* = 98 of 108 female fish) had *G. holbrooki* fin ray counts (Table 4); surprisingly, less than 10% (*n* = 10/108) had hybrid fin ray counts (Table 4). Of these, 10 females with hybrid fin ray counts, nine had semiprivate alleles from both species and one had western semiprivate alleles only (and fell into the western cluster) (Table 4).

**Figure 6:**
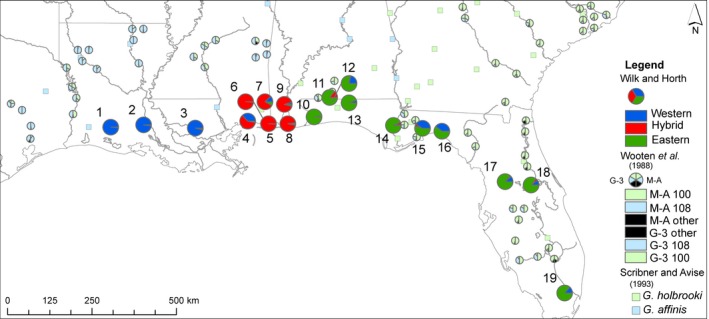
Microsatellite data from this study (Wilk and Horth), historical allozyme (Wooten et al., 1988) and mtDNA (Scribner & Avise, 1993) patterns for mosquito fish. Bright blue, red, and green pies represent Wilk and Horth's microsatellite alleles (western, hybrid, and eastern, respectively). Pale blue, green, and black pies represent Wooten et al. (1988) allozymes. For Wooten et al.'s (1988) allozymes, the left half of each pie represents the frequency of Glycerol‐3‐phosphate dehydrogenase (G‐3) alleles; the right half, M‐aspartate amino transferase (M‐A) alleles. Alleles are color‐coded for the most common allele by geographic region. G‐3 117 is represented by blue coloration on the left half of the pie, since it was common in the west. G‐3 100 is represented by green coloration on the left half of the pie since it was common in the east. M‐A 108 is blue on the right half of the pie since it was common in the west. M‐A 100 is green and was common in the east. Rare alleles are black. Pale blue and green squares represent Scribner and Avise's (1993) mtDNA for *G. affinis* and *G. holbrooki,* respectively

In the western cluster, all fish had *G. affinis* genotypes and phenotypes (Figures 5 and 6) except four fish. These had hybrid fin counts but 100% western cluster alleles.

Again, like for the genetic data, if we dissect the hybrid cluster, we see some interesting results: In the hybrid population furthest west (4‐MA), most females (78%) had western fin counts, fewer females had hybrid counts (17%), and very few (4%) had eastern counts. Genetically, however, nearly half of these fish (48%) contained a mix of western and hybrid cluster alleles (~50% each), about a third (30%) actually had only hybrid cluster alleles, and about a fifth (22%) had western cluster alleles. Male fin counts were also largely western (86%), less eastern (14%). Genetically, these males were also mixed hybrid and western cluster alleles (50% of each). So in this population, western fin counts were dominant, but hybrid alleles abounded.

However, in the middle of the hybrid cluster (the four most central cluster populations, 5‐MB to 8‐AB), the dominant fin count for females was eastern (88%); the remaining fish had an equal frequency of western and hybrid (6%) fin counts. Yet genetically, all of these females had hybrid genotypes. The majority of males in this region (87%) also had eastern fin counts, although a few were western (13%). Yet all of them had hybrid genotypes. Thus, eastern fin counts dominated most of the hybrid zone, despite hybrid genotypes.

In the population on the eastern edge of the hybrid cluster (9‐AC in AL), most females (84%) had western fin counts, with 11% hybrid and 5% eastern fin counts. Yet, (61%) of these fish had hybrid cluster genotypes. Just over one‐third of these fish (39%) had mixed hybrid (about half hybrid, and half eastern or half western) alleles. The males in this population all had western count fins, but were of mixed genetic origin (38% hybrid cluster alleles, 31% mixed hybrids, 23% western cluster, and 8% eastern cluster). Here, the western fin count was most abundant, again despite the high frequency of hybrid cluster genetics.

In the eastern cluster populations, nearly all (91%) females had eastern fin counts, but a few (9%) had hybrid counts. Most females (83%) were comprised of eastern alleles, but some were mixed hybrids (17%). Males here all had eastern fin phenotypes, except for one fish in AL; these males had mostly (67%) eastern cluster alleles, but some (33%) were mixed hybrids.

### 3.3 Comparison of present patterns to historical patterns

This work builds upon historical genetic and phenotypic work to demonstrate a hybrid zone for mosquito fish in the region where abrupt genotypic discontinuity has been previously identified over decades (Scribner & Avise, 1993; Wooten et al., 1988; Figure 6). The edges of the hybrid zone appear grossly similar to historical accounts with perhaps a slightly higher frequency of hybrid alleles a bit further west at the western end of the hybrid region, and a higher frequency of *G. holbrooki* alleles a bit further into the hybrid zone on the eastern edge, than in the past (Figure 6).

The classification of these fishes has been challenged since they were named and this analysis helps to explain why. Abrupt genetic discontinuities are evident, and phenotypic (fin counts) patterns do not match genetic ones closely for hybrids. This point is important relative to historical data for comparative purposes, but also because these “species” are frequently introduced for mosquito abatement and species identification has occurred by fin count and geographic origin.

Angus and Howell's (1996) fin ray data showed higher frequencies of hybrid counts in panhandle and western FL, into MS and AL than seen today (Figure 5). More *G. holbrooki* counts, and less hybrids, appear now (Figure 5). Hybrid counts were more common in some hybrid zone populations dominated by *G. holbrooki* and there were fewer hybrid counts in populations that were dominated by *G. affinis* (Figure 5). In the eastern part of the hybrid zone, we now find populations entirely *G. holbrooki* by fin count, where nearby populations had historically had hybrids, and moving westward in the zone, there appear to be more hybrid (or *G. holbrooki*) counts present in populations dominated by *G. affinis* (Figure 5). It is also relevant that in the western part of the hybrid zone, sometimes the *G. affinis* fin count dominates when genotypically fish are hybrids.

### 3.4 Hurricanes impacting the hybrid region

Analysis of NOAA data demonstrates that storms impacting the Gulf of Mexico and Western Atlantic Ocean have increased in frequency over the past century (*r* ^2^ = .668, *p* = .002), with ~67% of the variance in storm frequency explained by time. The frequency of intense hurricanes (categories 3–5) has doubled, up from 17 events in the 1980s, to 35 in the 2000s. The decade from 2000 to 2010 had the highest record of intense hurricanes since the 1900s. The vast majority of storms hit the Gulf of Mexico from the southeast, with wind gusts and flooding moving northwesterly. Since 1985, seven major storms, including Hurricanes Ivan, Andrew, Katrina, Dennis, Opal, Elena, and Jeanne, have substantially impacted the hybrid region. Empirical data demonstrate the movement of *G. holbrooki* during hurricanes (Caillouët, Carlson, Wesson, & Jordan, 2008).

## 4 Discussion

Three decades have elapsed since comprehensive allozyme work drew Wooten et al. (1988) to conclude that *G. affinis* and *G. holbrooki* should be considered two separate species with interspecific hybridization occurring in, but not limited to, the Mobile Bay drainage (Smith, Scribner, Hernandez, & Wooten, 1989). Here, using microsatellites we show three distinct genetic clusters of fish, suggesting western mosquito fish *(G. affinis)* persist in LA, hybrid fish are found in MS and much of southern AL, and eastern mosquito fish *(G. holbrooki)* are found in southern‐central AL, and throughout FL. The region where hybrid cluster fish are found is largely similar geographically to that which has been identified previously a number of times as a region of intergradation. The hybrid cluster is currently substantially different from the two parental populations, and distinct differences exist for the most easterly and westerly populations of the hybrid cluster, relative to the other hybrid populations, with a large proportion of eastern and western alleles present in the peripheral populations of the hybrid cluster.

Our results also support a hierarchical island model over an isolation by distance model, suggesting that demes within neighborhoods exchange more migrants than they do with other neighborhoods (Wright, 1943) which could potentially ultimately result in speciation. Historical connectivity, allowing for ancestrally shared alleles and interbreeding, resulting from historical biogeographic events could explain why the eastern and western species show higher connectivity to each other than to hybrid fish, especially if hybrids preferentially mate with one another, demonstrate hybrid vigor on incompatibility for some crosses, have evolved more recently, or are geographically isolated from the pure species to some degree.

Recurrent changes in sea level have clearly impacted freshwater fish populations. When sea level rose historically, the only fresh water habitat was a few points of higher elevation, meaning some reproductive isolation could have occurred for extant subpopulations, producing semiprivate alleles. Once seas receded, if neighborhood‐level gene flow was the model of movement, subsets private alleles may have migrated to novel local habitat. Since FL was almost twice the size it is today, the relatively greater landmass and expansive habitat near historic refuge may have contributed to the high number of private alleles arising. If we had sampled additional *G. affinis* territory, we may have found more private alleles in this species, too.

*Gambusia holbrooki* populations were shown to have higher heterozygosity than *G. affinis* previously which remains true today, and can contribute to fitness advantages (Orr, 2009). The relatively high heterozygosity in the east and the discontinuity between true species and the hybrid region that we identified are also consistent with previous work (Dr = 0.443 between the east and west, Wooten et al., 1988). The relatively low heterozygosity in the hybrid cluster, particularly in the western end, could support recent expansion of the hybrid region, nonrandom mating within hybrids, and/or some level of mating incompatibility. The relatively high degree of differentiation (pairwise *F*_ST_) between MS (5MB) and populations of western fish in LA, combined with the fact that this MS population has the lowest heterozygosity of all populations studied, indicates reduced gene flow between these regions relative to others, which is important with respect to understanding hybrid zone boundaries.

Genetic structure within species across mosquito fish populations has previously been reported to be fairly low (e.g., *F*_ST_ = 0.135 and 0.178), which is less than this current hybrid region, but also consistent with our overall results (Hernandez‐Martich & Smith, 1990; McClenaghan, Smith, & Smith, 1985). Genetic divergence between *G. affinis* and hybrids that are dominated by *G. holbrooki* alleles may be occurring right now in our study area which makes these data valuable for future research. Prior work (Lydeard, Wooten, & Smith, 1991) demonstrated greater structure (*F*_ST_ = 0.490) between *G. affinis* and *G. holbrooki,* so understanding more about this region is timely.

Fin ray counts were largely consistent with microsatellite data for the eastern and western genetic fish clusters or species *(G. affinis* and *G. holbrooki)*; however, counts were poorly correlated with genetic data in the hybrid region. Individuals with alleles from both species had fin ray phenotypes that could not be predicted based upon semiprivate alleles, or genetic match to cluster. Males, even those that had hybrid alleles, tended to have eastern mosquito fish fin counts, but not always. Previous work indicated that when *G. holbrooki* and *G. affinis* were crossed, F_1_ fish only had *G. holbrooki* fin ray counts, leading Hubbs, 1955 to propose Mendelian inheritance and dominance for ray count control. Hybrid fin counts only occurred after generations of backcrossing. However, Angus and Howell (1996) did not support that conjecture entirely and found that *G. holbrooki* fin ray counts did not dominate in hybrid populations. They also found evidence of nonrandom segregation, also supported by Scribner and Avise (1993). In the central four of our six hybrid zone populations, *G. holbrooki* fin ray counts dominate, despite hybrid genetics, which continues to suggest some possible form of dominance and confirms the relevant alleles associated with this trait may be acquired during backcrosses (or not lost). However, in one population genetically comprised largely of western and hybrid alleles, the western fin phenotype did dominate. This could suggest that fin count is polygenic and/or controlled by a form of incomplete dominance, where dominant alleles are found more often in the “true” species, with the *G. holbrooki* phenotype dominant over *G. affinis*. Future phenotypic research on fins should include comprehensive assessment of ventral and dorsal counts (see Angus & Howell, 1996) and minor gonopodial teeth differences that have been associated with species (Black & Howell, 1979) and were not evaluated here. Fin ray count data has helped determine species migration patterns and the frequency of species in geographic regions (Angus & Howell, 1996), so it is important to recognize that fin ray counts may not be reliable for assessing hybrids in the populations we studied.

Mating studies to determine compatibility between hybrid cluster fish, crossed with eastern and western fish, as well as crosses of “pure” species to determine which crosses are now viable and what quantity of progeny are produced, are in order. A moderate number of semiprivate alleles, and relatively low heterozygosity, were identified in the hybrid fish, suggesting some lack of successful breeding between cluster populations, which may align with Angus and Howell's (1996) thinking about nonrandom mix of these species.

The presence of a large heterogametic sex chromosome pair in *G. affinis* females was previously identified as causing some reproductive isolation between the two pure species. This may numerically favor crosses involving *G. holbrooki* females, where all F_1_ offspring are viable (Black & Howell, 1979). Thus, it would be interesting to assess the frequency of *G. holbrooki* mtDNA throughout the hybrid zone now, for comparison with historic frequencies identified by Scribner and Avise (1993).

Mate choice studies with hybrid and pure species would also determine whether mating preferences exist within versus across clusters now, specifically focusing on whether hybrids will mate preferentially with other hybrids. Then, evaluation of the production of multiple generations of hybrid crosses, for comparison to hybrid X pure species crosses to assess relative reproductive success, may yield data valuable for understanding the difference in the central populations of the hybrid region relative to the peripheral ones. This is particularly relevant since dramatic cross differences have been documented in the past.

The hybridization of *Gambusia* species is similar to that of tilapia, swordtails, and mollies: when female *G. holbrooki* and male *G. affinis* are crossed, all F_1_ offspring are male as a result of heterogamy. Some *G. holbrooki* alleles may be lost; these hybrids are also not as fit as pure *G. holbrooki*. In the reverse cross (female *G. affinis *× male *G. holbrooki*), a male bias also results, but not all (but 50–75%) progeny are male. These males may survive in the hybrid region but might not successfully colonize further east because they are outcompeted by pure *G. holbrooki* (Lucas & Southgate, 2012; Schultheis et al., 2009; Scribner & Avise, 1993). The impact of heterogamy, the production of nonviable offspring from some interspecific crosses, and genetically biased backcrosses could contribute to the hybrid regions relatively low heterozygosity. The greater viability of offspring from *G. holbrooki* females, some preference for larger males (Bisazza & Marin, 1991), or evolution from *G. holbrooki* could contribute to explaining why private alleles in the hybrid region appear to be more similar to *G. holbrooki* alleles, differing by only a few additions or deletions.

The combined genotypic and phenotypic data support a true hybrid region, currently with introgression, although the potential for incipient speciation exists. In the hybrid region, alleles and phenotypes still present from both species and AMOVA results indicate that most (83%) genetic variation is explained within populations, with a much smaller amount (6.9% and 9.5%) explained at the population or group level (respectively). Semiprivate alleles from both species are found in the hybrid region and there are fewer alleles overall in the hybrid region. However, identifying whether reproductive isolation exists between any clusters remains warranted since even single genes, adaptive in two parental species, can drive hybrid inviability (Presgraves, Balagopalan, Abmayr, & Orr, 2003).

Regarding future directions, climate change has impacted coastal freshwater systems in the past 30 years through sea level rise and increasing temperatures, which have forced cool water species, like catfish (*Ictalurus punctatus*), populations to move inland, or upstream, while potentially invasive warm water fish, like topminnows and sunfishes (*Fundulus* sp. and *Lepomis* sp.), have expanded their ranges (Mandrak, 1989; McCauley & Beitinger, 1992; Rahel & Olden, 2008). Recent hurricane prediction models indicate an increase in storm intensity in the western Atlantic Ocean (Bender et al., 2010) and NOAA data indicate that storms potentially impacting the mosquito fish hybrid zone have increased in frequency and intensity over the past century. Hurricanes affecting the region originate from the southeast then move in a northwesterly direction, inland. After Hurricane Katrina, thousands of swimming pools around New Orleans, LA, were colonized by multiple (*n* = 11) fish species, *Gambusia* being the most abundant by far (76% of the total number of fishes in the pools, Caillouët et al., 2008). Following two hurricanes (Frances and Jeanne) in 2004, *G. holbrooki* exhibited a twofold increase in the St. Sebastian River, FL (Paperno et al., 2006). Major hurricanes Ivan and Dennis (2004 and 2005, respectively) penetrated the hybrid region around Mobile Bay, which caused inundation of low lying areas, and connected oceanic to fresh water (Morgan & Sallenger, 2009a, 2009b). The impact of the directionality and frequency of hurricanes to movement of these invasive fishes from near coastal to inland populations warrants further study, especially given their ability to annihilate native species (Pyke, 2008).

Lastly, it is worth mentioning that humans contribute to mosquito fish movement. Frequent introductions for biological control of mosquitoes has occurred (Rupp, 1996) and ≥65 populations were established in MS by as early as 1919 (Ross & Brenneman, 2001). More recently, federal government, state agencies, biocontrol associations, and private citizens have introduced mosquito fish outside of their native range, to different continents, into irrigation canals, ditches, ponds, lakes, and swimming pools. The phenotypes of the two species are so similar and are not species specific, so there is high likelihood of mistaken identity and unintentional introduction of the wrong species or of hybrids to a given site. The findings discussed in this work are valuable as basic advances regarding our heuristic comprehension of hybrid zone dynamics but also as an important contribution to our conservation‐related knowledge on issues associated with breeding, stocking, and selling fast growing, highly invasive species, such as these phenotypic twins.

## Conflict of Interest

None declared.
