# Supplementary material for: A genetically distinct hybrid zone occurs for two globally invasive mosquito fish species with striking phenotypic resemblance
Source: Ecol Evol. 2016 Oct 24;6(23):8375–88. doi: 10.1002/ece3.2562 (PMC5167044; doi:10.1002/ece3.2562)
Supplement: Supplementary file 1 [file ECE3-6-8375-s001.docx]

Supporting Documents

Appendix S1. *G. affinis* microsatellite primers from Spencer *et al.* 1999.

| Locus | Primer+Sequence | Dye | GenBank+Number |
| --- | --- | --- | --- |
| Gafμ+1 | TCTGTTAGTGCTCAGCTGCAA | HEX | AF060919 |
|  | ATCAACAGCAGCCTCCTTCT |  |  |
| Gafμ+2 | CTCCAAACACACGTCCAATAATC | FAM | AF060920 |
|  | AGTTTCCCCAGCCGTTCAT |  |  |
| Gafμ+3 | CTCAGCCGTCATTTAGTCTCAT | FAM | AF060915 |
|  | GCACATAACATGGAAACAGTAAAC |  |  |
| Gafμ+4 | ACAACGGAGACCTGCTGGAGTGG | FAM | AF060914 |
|  | CGCGAACCGTCCGTTATCCGTA |  |  |
| Gafμ+6 | ACGAAGAGAGCAGCGGACTTTTGG | HEX | AF060917 |
|  | CGCCGGACAGACCAGCCTCA |  |  |
| Gafμ+7 | CACAGAACAACACAGAAACTGGAGG | HEX | AF060918 |

TGCCGATGGATGTTCCTGTTAG

Appendix S2. Summary of genetic diversity data from 19 populations in the SE US for each of 6 loci. N= number of individuals, #= number of alleles, R= allele range, HO= observed heterozygosity, He= expected heterozygosity, AR= allelic richness adjusted for smallest population, and FIs= inbreeding coefficient

| Locus | | N | # | R | HO | HE HWE | | AR | Fis | Locus | | N | # | R | HO | HE | HWE | AR | Fis |
| --- | --- | --- | --- | --- | --- | --- | --- | --- | --- | --- | --- | --- | --- | --- | --- | --- | --- | --- | --- |
| 1LB | Gafu 1* | 31 | 1 | 1 - | - | - |  | 1.000 | NA | 11AD | Gafu 1 | 14 | 4 | 12 | 0.714 | 0.749 | 0.110 | 4.000 | 0.048 |
|  | Gafu 2 | 31 | 11 | 46 | 0.770 | 0.834 | 0.168 | 8.646 | 0.103 |  | Gafu 2 | 14 | 7 | 38 | 0.857 | 0.767 | 0.613 | 7.000 | -0.122 |
|  | Gafu 3 | 31 | 15 | 53 | 0.844 | 0.885 | 0.451 | 11.843 | 0.048 |  | Gafu 3 | 14 | 7 | 73 | 0.714 | 0.730 | 0.816 | 7.000 | 0.023 |
|  | Gafu 4 | 31 | 13 | 76 | 0.875 | 0.878 | 0.961 | 9.956 | 0.003 |  | Gafu 4 | 14 | 10 | 80 | 1.000 | 0.886 | 0.503 | 10.000 | -0.134 |
|  | Gafu 6 | 31 | 5 | 20 | 0.625 | 0.647 | 0.136 | 4.373 | 0.035 |  | Gafu 6 | 14 | 6 | 15 | 0.643 | 0.677 | 0.722 | 6.000 | 0.053 |
|  | Gafu 7 | 31 | 12 | 42 | 0.969 | 0.864 | 0.177 | 9.229 | -0.123 |  | Gafu 7 | 14 | 7 | 50 | 0.786 | 0.788 | 0.160 | 7.000 | 0.003 |
| 2LA | Gafu 1 | 30 | 2 | 2 | 0.067 | 0.066 | 1.000 | 1.720 | -0.018 | 12HL | Gafu 1 | 30 | 4 | 8 | 0.700 | 0.673 | 0.015 | 3.855 | -0.041 |
|  | Gafu 2 | 30 | 12 | 40 | 0.733 | 0.795 | 0.064 | 9.013 | 0.079 |  | Gafu 2 | 30 | 11 | 38 | 0.767 | 0.838 | 0.784 | 8.265 | 0.087 |
|  | Gafu 3 | 30 | 12 | 44 | 0.767 | 0.887 | 0.014 | 10.273 | 0.138 |  | Gafu 3 | 30 | 13 | 51 | 0.900 | 0.887 | 0.462 | 10.408 | -0.015 |
|  | Gafu 4 | 30 | 14 | 69 | 0.900 | 0.849 | 0.107 | 10.770 | -0.062 |  | Gafu 4 | 30 | 14 | 103 | 0.833 | 0.884 | 0.095 | 10.593 | 0.058 |
|  | Gafu 6 | 30 | 7 | 20 | 0.633 | 0.592 | 0.760 | 5.577 | -0.072 |  | Gafu 6 | 30 | 7 | 15 | 0.600 | 0.795 | 0.036 | 6.267 | 0.249 |
|  | Gafu 7 | 30 | 17 | 50 | 0.900 | 0.908 | 0.708 | 12.475 | 0.009 |  | Gafu 7 | 30 | 9 | 24 | 0.733 | 0.884 | 0.290 | 8.322 | 0.173 |
| 3LC | Gafu 1 | 29 | 3 | 9 | 0.172 | 0.163 | 1.000 | 2.418 | -0.061 | 13KL | Gafu 1 | 32 | 4 | 8 | 0.719 | 0.726 | 0.734 | 3.987 | 0.010 |
|  | Gafu 2 | 29 | 13 | 40 | 0.655 | 0.782 | 0.011 | 9.527 | 0.165 |  | Gafu 2 | 32 | 6 | 20 | 0.531 | 0.600 | 0.147 | 5.329 | 0.116 |
|  | Gafu 3 | 29 | 13 | 48 | 0.931 | 0.906 | 0.281 | 10.832 | -0.029 |  | Gafu 3 | 32 | 11 | 40 | 0.875 | 0.870 | 0.122 | 8.825 | -0.006 |
|  | Gafu 4 | 29 | 16 | 103 | 0.793 | 0.911 | 0.178 | 12.137 | 0.131 |  | Gafu 4 | 32 | 13 | 128 | 0.688 | 0.845 | 0.124 | 10.164 | 0.189 |
|  | Gafu 6 | 29 | 6 | 20 | 0.517 | 0.527 | 0.626 | 4.897 | 0.019 |  | Gafu 6 | 32 | 7 | 15 | 0.719 | 0.813 | 0.259 | 6.463 | 0.117 |
|  | Gafu 7 | 29 | 13 | 29 | 0.724 | 0.859 | 0.058 | 10.715 | 0.159 |  | Gafu 7 | 32 | 10 | 35 | 0.938 | 0.839 | 0.560 | 8.229 | -0.120 |
| 4MA | Gafu 1 | 31 | 4 | 13 | 0.355 | 0.378 | 0.541 | 3.292 | 0.063 | 14FA | Gafu 1 | 30 | 5 | 10 | 0.433 | 0.414 | 0.198 | 3.788 | -0.049 |
|  | Gafu 2 | 31 | 10 | 32 | 0.774 | 0.774 | 0.929 | 7.548 | -0.001 |  | Gafu 2 | 30 | 10 | 32 | 0.533 | 0.602 | 0.051 | 7.083 | 0.115 |
|  | Gafu 3 | 31 | 14 | 77 | 0.935 | 0.902 | 0.912 | 10.681 | -0.038 |  | Gafu 3 | 30 | 13 | 53 | 0.833 | 0.785 | 0.060 | 9.082 | -0.062 |
|  | Gafu 4 | 31 | 15 | 89 | 0.903 | 0.879 | 0.704 | 11.532 | -0.028 |  | Gafu 4 | 30 | 17 | 89 | 0.867 | 0.942 | 0.118 | 13.920 | 0.081 |
|  | Gafu 6 | 31 | 6 | 16 | 0.871 | 0.803 | 0.916 | 5.429 | -0.086 |  | Gafu 6 | 30 | 5 | 13 | 0.233 | 0.579 | **0.000** | 4.388 | 0.601 |
|  | Gafu 7 | 31 | 13 | 70 | 0.806 | 0.829 | 0.672 | 9.972 | 0.028 |  | Gafu 7 | 30 | 12 | 46 | 0.733 | 0.772 | 0.235 | 8.213 | 0.051 |
| 5MB | Gafu 1 | 30 | 3 | 6 | 0.067 | 0.066 | 1.000 | 1.933 | -0.009 | 15WA | Gafu 1 | 30 | 4 | 8 | 0.567 | 0.479 | 0.929 | 3.701 | -0.187 |
|  | Gafu 2 | 30 | 5 | 16 | 0.267 | 0.245 | 1.000 | 3.363 | -0.089 |  | Gafu 2 | 30 | 16 | 42 | 0.867 | 0.894 | 0.156 | 12.145 | 0.031 |
|  | Gafu 3 | 30 | 17 | 135 | 0.867 | 0.902 | 0.157 | 12.113 | 0.040 |  | Gafu 3 | 30 | 13 | 53 | 0.900 | 0.911 | 0.134 | 11.178 | 0.013 |
|  | Gafu 4 | 30 | 18 | 97 | 0.867 | 0.888 | 0.565 | 12.446 | 0.024 |  | Gafu 4 | 30 | 16 | 107 | 0.900 | 0.927 | 0.253 | 12.686 | 0.029 |
|  | Gafu 6 | 30 | 4 | 9 | 0.233 | 0.273 | 0.326 | 3.319 | 0.147 |  | Gafu 6 | 30 | 7 | 15 | 0.433 | 0.802 | **0.000** | 6.421 | 0.464 |
|  | Gafu 7 | 30 | 13 | 75 | 0.633 | 0.610 | 0.759 | 8.334 | -0.039 |  | Gafu 7 | 30 | 14 | 46 | 0.933 | 0.850 | 0.157 | 9.936 | -0.100 |
| 6MC | Gafu 1 | 31 | 3 | 13 | 0.097 | 0.095 | 1.000 | 2.155 | -0.023 | 16FB | Gafu 1 | 30 | 5 | 10 | 0.500 | 0.459 | 0.440 | 3.896 | -0.092 |
|  | Gafu 2 | 31 | 4 | 26 | 0.419 | 0.467 | 0.053 | 3.680 | 0.104 |  | Gafu 2 | 30 | 15 | 44 | 0.933 | 0.877 | 0.400 | 11.477 | -0.066 |
|  | Gafu 3 | 31 | 15 | 135 | 0.968 | 0.918 | 0.610 | 11.730 | -0.055 |  | Gafu 3 | 30 | 14 | 57 | 0.733 | 0.873 | 0.047 | 10.116 | 0.163 |
|  | Gafu 4 | 31 | 16 | 68 | 0.839 | 0.902 | 0.310 | 11.686 | 0.071 |  | Gafu 4 | 30 | 19 | 86 | 0.900 | 0.936 | 0.195 | 14.224 | 0.039 |
|  | Gafu 6 | 31 | 6 | 16 | 0.355 | 0.343 | 0.472 | 4.288 | -0.034 |  | Gafu 6 | 30 | 7 | 20 | 0.567 | 0.794 | 0.016 | 5.892 | 0.290 |
|  | Gafu 7 | 31 | 12 | 59 | 0.548 | 0.495 | 0.907 | 7.503 | -0.110 |  | Gafu 7 | 30 | 11 | 25 | 0.900 | 0.890 | 0.742 | 9.549 | -0.011 |
| 7AA | Gafu 1 | 32 | 2 | 2 | 0.219 | 0.198 | 1.000 | 1.987 | -0.107 | 17PA | Gafu 1 | 30 | 3 | 6 | 0.433 | 0.594 | 0.020 | 3.000 | 0.274 |
|  | Gafu 2 | 32 | 4 | 22 | 0.531 | 0.534 | 0.092 | 3.431 | 0.006 |  | Gafu 2 | 30 | 11 | 20 | 0.933 | 0.876 | 0.385 | 9.153 | -0.067 |
|  | Gafu 3 | 32 | 11 | 73 | 0.813 | 0.870 | 0.172 | 8.877 | 0.067 |  | Gafu 3 | 30 | 17 | 66 | 0.900 | 0.914 | 0.393 | 12.698 | 0.016 |
|  | Gafu 4 | 32 | 13 | 76 | 0.656 | 0.875 | **0.000** | 9.899 | 0.253 |  | Gafu 4 | 30 | 15 | 70 | 0.867 | 0.916 | 0.113 | 12.068 | 0.055 |
|  | Gafu 6 | 32 | 5 | 13 | 0.219 | 0.206 | 1.000 | 3.391 | -0.061 |  | Gafu 6 | 30 | 8 | 18 | 0.400 | 0.771 | **0.000** | 6.608 | 0.485 |
|  | Gafu 7 | 32 | 12 | 64 | 0.906 | 0.887 | 0.370 | 10.323 | -0.022 |  | Gafu 7 | 30 | 14 | 38 | 0.800 | 0.907 | 0.355 | 11.287 | 0.120 |
| 8AB | Gafu 1 | 32 | 2 | 6 | 0.125 | 0.119 | 1.000 | 1.907 | -0.051 | 18WE | Gafu 1 | 27 | 5 | 10 | 0.407 | 0.415 | 0.504 | 4.542 | 0.019 |
|  | Gafu 2 | 32 | 5 | 48 | 0.156 | 0.151 | 1.000 | 3.000 | -0.037 |  | Gafu 2 | 27 | 15 | 42 | 0.852 | 0.883 | 0.440 | 11.348 | 0.035 |
|  | Gafu 3 | 32 | 14 | 84 | 0.835 | 0.916 | 0.379 | 11.952 | 0.080 |  | Gafu 3 | 27 | 12 | 45 | 0.926 | 0.897 | 0.456 | 10.306 | -0.033 |
|  | Gafu 4 | 32 | 12 | 84 | 0.563 | 0.741 | 0.008 | 8.969 | 0.244 |  | Gafu 4 | 27 | 17 | 70 | 0.815 | 0.932 | 0.080 | 13.498 | 0.128 |
|  | Gafu 6 | 32 | 4 | 13 | 0.469 | 0.500 | 0.459 | 3.411 | 0.063 |  | Gafu 6 | 27 | 8 | 20 | 0.630 | 0.856 | 0.006 | 7.552 | 0.268 |
|  | Gafu 7 | 32 | 12 | 40 | 0.813 | 0.798 | 0.011 | 8.447 | -0.019 |  | Gafu 7 | 27 | 11 | 40 | 0.704 | 0.761 | 0.490 | 8.973 | 0.077 |
| 9AC | Gafu 1 | 31 | 3 | 12 | 0.097 | 0.153 | 0.159 | 2.368 | 0.371 | 19MI | Gafu 1 | 28 | 6 | 12 | 0.857 | 0.764 | 0.599 | 4.999 | -0.125 |
|  | Gafu 2 | 31 | 8 | 28 | 0.613 | 0.746 | 0.248 | 6.618 | 0.180 |  | Gafu 2 | 28 | 16 | 54 | 0.821 | 0.932 | 0.209 | 13.152 | 0.120 |
|  | Gafu 3 | 31 | 18 | 105 | 0.968 | 0.905 | 0.529 | 12.436 | -0.070 |  | Gafu 3 | 28 | 14 | 53 | 0.786 | 0.915 | 0.071 | 11.658 | 0.143 |
|  | Gafu 4 | 31 | 15 | 81 | 0.935 | 0.909 | 0.138 | 12.409 | -0.030 |  | Gafu 4 | 28 | 19 | 77 | 0.714 | 0.945 | **0.000** | 14.697 | 0.248 |
|  | Gafu 6 | 31 | 5 | 10 | 0.452 | 0.610 | 0.059 | 4.408 | 0.263 |  | Gafu 6 | 28 | 8 | 20 | 0.714 | 0.823 | 0.196 | 6.885 | 0.135 |
|  | Gafu 7 | 31 | 13 | 35 | 0.710 | 0.759 | 0.946 | 9.082 | 0.066 |  | Gafu 7 | 28 | 13 | 42 | 0.821 | 0.906 | 0.129 | 10.895 | 0.095 |
| 10ES | Gafu 1 30 4 12 0.767 0.752 0.813 4.000 -0.020 | | | | | | | | |  | | | | | | | | | |
|  | Gafu 2 30 10 54 0.833 0.857 0.349 8.189 0.027 | | | | | | | | |  |  |  |  |  |  |  |  |  |  |
|  | Gafu 3 30 8 75 0.800 0.808 0.516 7.084 0.010 | | | | | | | | |  |  |  |  |  |  |  |  |  |  |
|  | Gafu 4 30 12 113 0.933 0.879 0.620 9.594 -0.064 | | | | | | | | |  |  |  |  |  |  |  |  |  |  |
|  | Gafu 6 30 7 15 0.567 0.775 0.038 5.786 0.272 | | | | | | | | |  |  |  |  |  |  |  |  |  |  |
|  | Gafu 7 30 7 50 0.800 0.810 0.805 6.796 0.012 | | | | | | | | |  |  |  |  |  |  |  |  |  |  |

Appendix S3. Microsatellite Data in STRUCTURE format.

| LB1 | 1 | 105 | 151 | 247 | 214 | 195 | 166 |
| --- | --- | --- | --- | --- | --- | --- | --- |
| LB1 | 1 | 105 | 151 | 250 | 221 | 197 | 168 |
| LB10 | 1 | 105 | 157 | 234 | 200 | 197 | 162 |
| LB10 | 1 | 105 | 161 | 250 | 221 | 201 | 185 |
| LB11 | 1 | 105 | 145 | 234 | 200 | 197 | 175 |
| LB11 | 1 | 105 | 161 | 234 | 214 | 197 | 177 |
| LB12 | 1 | 105 | 151 | 218 | 191 | 195 | 162 |
| LB12 | 1 | 105 | 157 | 244 | 200 | 201 | 177 |
| LB13 | 1 | 105 | 161 | 237 | 207 | 195 | 175 |
| LB13 | 1 | 105 | 161 | 244 | 221 | 197 | 175 |
| LB14 | 1 | 105 | 159 | 225 | 185 | 181 | 166 |
| LB14 | 1 | 105 | 161 | 264 | 240 | 197 | 168 |
| LB15 | 1 | 105 | 161 | 247 | 221 | 197 | 162 |
| LB15 | 1 | 105 | 161 | 250 | 221 | 201 | 185 |
| LB16 | 1 | 105 | 157 | 250 | 200 | 197 | 162 |
| LB16 | 1 | 105 | 161 | 250 | 211 | 197 | 179 |
| LB17 | 1 | 105 | 161 | 250 | 217 | 195 | 175 |
| LB17 | 1 | 105 | 161 | 250 | 217 | 195 | 177 |
| LB18 | 1 | 105 | 151 | 244 | 185 | 197 | 175 |
| LB18 | 1 | 105 | 165 | 250 | 214 | 201 | 185 |
| LB19 | 1 | 105 | 161 | 261 | 200 | 195 | 168 |
| LB19 | 1 | 105 | 165 | 264 | 207 | 201 | 171 |
| LB2 | 1 | 105 | 145 | 218 | 191 | 195 | 162 |
| LB2 | 1 | 105 | 147 | 268 | 217 | 197 | 175 |
| LB20 | 1 | 105 | 145 | 240 | 185 | 195 | 162 |
| LB20 | 1 | 105 | 161 | 258 | 191 | 195 | 168 |
| LB21 | 1 | 105 | 151 | 250 | 185 | 195 | 162 |
| LB21 | 1 | 105 | 159 | 254 | 185 | 195 | 181 |
| LB22 | 1 | 105 | 151 | 234 | 221 | 197 | 175 |
| LB22 | 1 | 105 | 159 | 271 | 261 | 197 | 185 |
| LB23 | 1 | 105 | 153 | 250 | 185 | 197 | 166 |
| LB23 | 1 | 105 | 157 | 250 | 221 | 197 | 168 |
| LB24 | 1 | 105 | 155 | 240 | 191 | 197 | 162 |
| LB24 | 1 | 105 | 169 | 250 | 217 | 201 | 175 |
| LB25 | 1 | 105 | 151 | 218 | 214 | 195 | 171 |
| LB25 | 1 | 105 | 155 | 250 | 221 | 197 | 175 |
| LB26 | 1 | 105 | 157 | 250 | 217 | 197 | 175 |
| LB26 | 1 | 105 | 159 | 271 | 221 | 197 | 177 |
| LB27 | 1 | 105 | 151 | 230 | 195 | 197 | 162 |
| LB27 | 1 | 105 | 161 | 240 | 214 | 201 | 175 |
| LB28 | 1 | 105 | 157 | 258 | 185 | 197 | 162 |
| LB28 | 1 | 105 | 169 | 271 | 203 | 201 | 175 |
| LB29 | 1 | 105 | 159 | 247 | 185 | 181 | 175 |
| LB29 | 1 | 105 | 161 | 271 | 217 | 195 | 177 |

| LB3 | 1 | 105 | 151 | 234 | 185 | 197 | 175 |
| --- | --- | --- | --- | --- | --- | --- | --- |
| LB3 | 1 | 105 | 159 | 240 | 221 | 197 | 185 |
| LB30 | 1 | 105 | 161 | 244 | 214 | 197 | 177 |
| LB30 | 1 | 105 | 165 | 258 | 217 | 201 | 181 |
| LB31 | 1 | 105 | 161 | 240 | 214 | 197 | 166 |
| LB31 | 1 | 105 | 161 | 250 | 221 | 197 | 175 |
| LB32 | 1 | 105 | 161 | 234 | 185 | 195 | 166 |
| LB32 | 1 | 105 | 161 | 254 | 200 | 197 | 175 |
| LB5 | 1 | 105 | 151 | 240 | 185 | 197 | 171 |
| LB5 | 1 | 105 | 165 | 268 | 207 | 201 | 177 |
| LB6 | 1 | 105 | 151 | 225 | 203 | 197 | 166 |
| LB6 | 1 | 105 | 159 | 234 | 221 | 197 | 177 |
| LB7 | 1 | 105 | 159 | 218 | 185 | 195 | 171 |
| LB7 | 1 | 105 | 159 | 244 | 221 | 197 | 177 |
| LB8 | 1 | 105 | 151 | 225 | 185 | 195 | 166 |
| LB8 | 1 | 105 | 151 | 250 | 224 | 197 | 175 |
| LB9 | 1 | 105 | 147 | 234 | 217 | 195 | 177 |
| LB9 | 1 | 105 | 159 | 237 | 221 | 197 | 193 |
| LA1 | 2 | 105 | 151 | 250 | 203 | 191 | 166 |
| LA1 | 2 | 105 | 161 | 250 | 203 | 197 | 185 |
| LA11 | 2 | 105 | 151 | 240 | 185 | 181 | 177 |
| LA11 | 2 | 105 | 151 | 250 | 224 | 197 | 183 |
| LA12 | 2 | 105 | 145 | 240 | 200 | 195 | 162 |
| LA12 | 2 | 105 | 153 | 254 | 203 | 197 | 177 |
| LA13 | 2 | 105 | 151 | 234 | 185 | 197 | 168 |
| LA13 | 2 | 105 | 151 | 274 | 254 | 201 | 191 |
| LA14 | 2 | 105 | 165 | 240 | 185 | 191 | 171 |
| LA14 | 2 | 105 | 169 | 250 | 191 | 197 | 179 |
| LA15 | 2 | 105 | 151 | 244 | 185 | 197 | 181 |
| LA15 | 2 | 105 | 155 | 250 | 211 | 197 | 183 |
| LA16 | 2 | 105 | 151 | 244 | 191 | 197 | 155 |
| LA16 | 2 | 105 | 151 | 264 | 207 | 197 | 191 |
| LA17 | 2 | 105 | 151 | 250 | 185 | 193 | 166 |
| LA17 | 2 | 105 | 157 | 250 | 185 | 201 | 171 |
| LA18 | 2 | 105 | 165 | 254 | 185 | 197 | 168 |
| LA18 | 2 | 105 | 169 | 254 | 191 | 197 | 181 |
| LA19 | 2 | 105 | 151 | 244 | 185 | 195 | 166 |
| LA19 | 2 | 105 | 151 | 261 | 191 | 197 | 181 |
| LA2 | 2 | 105 | 151 | 234 | 185 | 188 | 175 |
| LA2 | 2 | 105 | 157 | 274 | 191 | 197 | 185 |
| LA20 | 2 | 105 | 151 | 254 | 185 | 195 | 166 |
| LA20 | 2 | 105 | 151 | 264 | 211 | 197 | 191 |
| LA21 | 2 | 105 | 165 | 244 | 185 | 197 | 166 |
| LA21 | 2 | 105 | 171 | 244 | 185 | 197 | 181 |
| LA22 | 2 | 105 | 151 | 240 | 191 | 195 | 162 |
| LA22 | 2 | 105 | 161 | 240 | 200 | 197 | 166 |

| LA23 | 2 | 105 | 147 | 240 | 185 | 191 | 181 |
| --- | --- | --- | --- | --- | --- | --- | --- |
| LA23 | 2 | 105 | 151 | 244 | 248 | 197 | 191 |
| LA25 | 2 | 105 | 161 | 240 | 228 | 197 | 162 |
| LA25 | 2 | 105 | 165 | 250 | 254 | 201 | 171 |
| LA26 | 2 | 105 | 151 | 230 | 214 | 197 | 181 |
| LA26 | 2 | 105 | 155 | 254 | 221 | 197 | 185 |
| LA27 | 2 | 105 | 161 | 240 | 185 | 191 | 193 |
| LA27 | 2 | 105 | 161 | 258 | 221 | 197 | 205 |
| LA28 | 2 | 105 | 151 | 240 | 217 | 181 | 183 |
| LA28 | 2 | 105 | 153 | 250 | 240 | 197 | 198 |
| LA29 | 2 | 105 | 151 | 254 | 185 | 191 | 183 |
| LA29 | 2 | 105 | 157 | 254 | 224 | 201 | 183 |
| LA3 | 2 | 103 | 155 | 244 | 207 | 191 | 166 |
| LA3 | 2 | 105 | 161 | 274 | 217 | 197 | 183 |
| LA30 | 2 | 105 | 151 | 234 | 203 | 197 | 164 |
| LA30 | 2 | 105 | 155 | 240 | 217 | 201 | 175 |
| LA31 | 2 | 105 | 161 | 234 | 185 | 197 | 175 |
| LA31 | 2 | 105 | 161 | 268 | 191 | 197 | 191 |
| LA32 | 2 | 105 | 151 | 261 | 185 | 197 | 164 |
| LA32 | 2 | 105 | 151 | 268 | 217 | 197 | 166 |
| LA4 | 2 | 105 | 157 | 250 | 185 | 197 | 175 |
| LA4 | 2 | 105 | 169 | 250 | 211 | 197 | 185 |
| LA5 | 2 | 105 | 151 | 230 | 203 | 191 | 173 |
| LA5 | 2 | 105 | 161 | 234 | 214 | 197 | 181 |
| LA6 | 2 | 105 | 131 | 247 | 185 | 197 | 166 |
| LA6 | 2 | 105 | 157 | 250 | 191 | 197 | 166 |
| LA7 | 2 | 105 | 147 | 247 | 200 | 191 | 166 |
| LA7 | 2 | 105 | 151 | 261 | 217 | 191 | 181 |
| LA8 | 2 | 103 | 161 | 234 | 185 | 197 | 166 |
| LA8 | 2 | 105 | 165 | 240 | 191 | 197 | 183 |
| LA9 | 2 | 105 | 159 | 240 | 191 | 195 | 181 |
| LA9 | 2 | 105 | 161 | 258 | 214 | 197 | 181 |
| LC10 | 3 | 105 | 147 | 247 | 195 | 195 | 166 |
| LC10 | 3 | 105 | 159 | 261 | 203 | 201 | 168 |
| LC11 | 3 | 105 | 157 | 240 | 200 | 197 | 175 |
| LC11 | 3 | 105 | 159 | 247 | 217 | 197 | 177 |
| LC12 | 3 | 105 | 151 | 247 | 185 | 195 | 166 |
| LC12 | 3 | 105 | 161 | 261 | 185 | 197 | 173 |
| LC13 | 3 | 105 | 153 | 254 | 200 | 195 | 166 |
| LC13 | 3 | 105 | 167 | 258 | 200 | 197 | 173 |
| LC14 | 3 | 103 | 151 | 240 | 191 | 197 | 166 |
| LC14 | 3 | 105 | 151 | 278 | 217 | 201 | 173 |
| LC15 | 3 | 103 | 139 | 234 | 200 | 191 | 171 |
| LC15 | 3 | 105 | 161 | 247 | 203 | 197 | 185 |
| LC17 | 3 | 105 | 159 | 230 | 185 | 197 | 166 |
| LC17 | 3 | 105 | 161 | 237 | 200 | 197 | 166 |

| LC19 | 3 | 105 | 151 | 250 | 185 | 197 | 179 |
| --- | --- | --- | --- | --- | --- | --- | --- |
| LC19 | 3 | 105 | 151 | 271 | 236 | 201 | 191 |
| LC2 | 3 | 105 | 143 | 240 | 195 | 197 | 166 |
| LC2 | 3 | 105 | 157 | 244 | 232 | 197 | 185 |
| LC20 | 3 | 105 | 151 | 247 | 203 | 197 | 181 |
| LC20 | 3 | 105 | 165 | 254 | 214 | 197 | 183 |
| LC21 | 3 | 105 | 151 | 240 | 207 | 197 | 181 |
| LC21 | 3 | 105 | 151 | 254 | 207 | 197 | 181 |
| LC22 | 3 | 105 | 143 | 234 | 191 | 193 | 166 |
| LC22 | 3 | 105 | 161 | 244 | 195 | 197 | 181 |
| LC23 | 3 | 105 | 151 | 237 | 200 | 197 | 166 |
| LC23 | 3 | 105 | 151 | 247 | 228 | 197 | 166 |
| LC24 | 3 | 105 | 157 | 250 | 214 | 197 | 164 |
| LC24 | 3 | 105 | 165 | 250 | 217 | 197 | 166 |
| LC25 | 3 | 105 | 155 | 230 | 200 | 197 | 179 |
| LC25 | 3 | 105 | 161 | 261 | 200 | 197 | 191 |
| LC26 | 3 | 103 | 151 | 234 | 185 | 197 | 173 |
| LC26 | 3 | 105 | 171 | 240 | 248 | 201 | 173 |
| LC27 | 3 | 105 | 151 | 240 | 185 | 197 | 166 |
| LC27 | 3 | 105 | 161 | 250 | 240 | 197 | 166 |
| LC28 | 3 | 105 | 151 | 240 | 185 | 195 | 181 |
| LC28 | 3 | 105 | 161 | 261 | 203 | 201 | 181 |
| LC29 | 3 | 96 | 151 | 240 | 203 | 197 | 164 |
| LC29 | 3 | 105 | 151 | 250 | 214 | 197 | 179 |
| LC3 | 3 | 105 | 139 | 264 | 185 | 181 | 166 |
| LC3 | 3 | 105 | 161 | 264 | 191 | 197 | 185 |
| LC30 | 3 | 105 | 151 | 237 | 191 | 191 | 166 |
| LC30 | 3 | 105 | 151 | 254 | 240 | 197 | 177 |
| LC31 | 3 | 105 | 131 | 247 | 185 | 191 | 162 |
| LC31 | 3 | 105 | 157 | 261 | 185 | 197 | 171 |
| LC32 | 3 | 103 | 151 | 247 | 195 | 197 | 166 |
| LC32 | 3 | 105 | 157 | 250 | 228 | 197 | 183 |
| LC4 | 3 | 105 | 151 | 230 | 217 | 195 | 175 |
| LC4 | 3 | 105 | 151 | 240 | 228 | 197 | 175 |
| LC5 | 3 | 105 | 151 | 237 | 214 | 191 | 175 |
| LC5 | 3 | 105 | 151 | 247 | 214 | 197 | 181 |
| LC6 | 3 | 105 | 155 | 234 | 185 | 191 | 166 |
| LC6 | 3 | 105 | 161 | 244 | 288 | 197 | 175 |
| LC7 | 3 | 105 | 151 | 244 | 207 | 197 | 166 |
| LC7 | 3 | 105 | 151 | 254 | 244 | 197 | 166 |
| LC8 | 3 | 105 | 151 | 240 | 200 | 191 | 177 |
| LC8 | 3 | 105 | 155 | 254 | 203 | 191 | 183 |
| LC9 | 3 | 105 | 165 | 237 | 203 | 197 | 183 |
| LC9 | 3 | 105 | 165 | 247 | 221 | 197 | 185 |

| MA11 | 4 | 109 | 129 | 237 | 221 | 191 | 171 |
| --- | --- | --- | --- | --- | --- | --- | --- |
| MA11 | 4 | 109 | 143 | 264 | 261 | 197 | 181 |
| MA12 | 4 | 105 | 129 | 244 | 203 | 191 | 162 |
| MA12 | 4 | 105 | 159 | 264 | 269 | 197 | 185 |
| MA13 | 4 | 105 | 143 | 234 | 214 | 185 | 162 |
| MA13 | 4 | 105 | 155 | 254 | 217 | 188 | 162 |
| MA14 | 4 | 105 | 155 | 244 | 214 | 191 | 166 |
| MA14 | 4 | 109 | 159 | 254 | 224 | 197 | 181 |
| MA15 | 4 | 105 | 129 | 247 | 224 | 185 | 168 |
| MA15 | 4 | 105 | 131 | 247 | 269 | 191 | 181 |
| MA16 | 4 | 105 | 143 | 247 | 180 | 191 | 171 |
| MA16 | 4 | 109 | 155 | 254 | 191 | 197 | 177 |
| MA17 | 4 | 105 | 131 | 244 | 200 | 185 | 185 |
| MA17 | 4 | 105 | 147 | 258 | 214 | 197 | 185 |
| MA18 | 4 | 105 | 129 | 237 | 224 | 185 | 162 |
| MA18 | 4 | 109 | 143 | 268 | 261 | 191 | 164 |
| MA19 | 4 | 105 | 145 | 234 | 214 | 185 | 160 |
| MA19 | 4 | 105 | 151 | 240 | 232 | 197 | 162 |
| MA2 | 4 | 105 | 129 | 240 | 214 | 185 | 162 |
| MA2 | 4 | 105 | 143 | 264 | 217 | 197 | 162 |
| MA20 | 4 | 105 | 131 | 225 | 207 | 191 | 168 |
| MA20 | 4 | 105 | 131 | 264 | 214 | 195 | 173 |
| MA21 | 4 | 105 | 143 | 247 | 214 | 197 | 162 |
| MA21 | 4 | 107 | 159 | 302 | 240 | 201 | 179 |
| MA22 | 4 | 105 | 143 | 237 | 200 | 195 | 162 |
| MA22 | 4 | 105 | 159 | 254 | 214 | 201 | 171 |
| MA23 | 4 | 105 | 143 | 234 | 224 | 195 | 162 |
| MA23 | 4 | 105 | 159 | 240 | 236 | 201 | 162 |
| MA24 | 4 | 105 | 143 | 237 | 214 | 191 | 162 |
| MA24 | 4 | 109 | 161 | 240 | 224 | 197 | 185 |
| MA25 | 4 | 105 | 143 | 234 | 207 | 191 | 162 |
| MA25 | 4 | 105 | 143 | 237 | 207 | 201 | 162 |
| MA26 | 4 | 105 | 143 | 240 | 203 | 197 | 173 |
| MA26 | 4 | 105 | 143 | 247 | 214 | 201 | 230 |
| MA27 | 4 | 105 | 139 | 247 | 214 | 195 | 177 |
| MA27 | 4 | 109 | 143 | 254 | 214 | 197 | 185 |
| MA28 | 4 | 96 | 143 | 244 | 214 | 185 | 166 |
| MA28 | 4 | 109 | 143 | 264 | 232 | 191 | 171 |
| MA29 | 4 | 96 | 143 | 237 | 191 | 185 | 162 |
| MA29 | 4 | 105 | 155 | 247 | 224 | 195 | 166 |
| MA3 | 4 | 105 | 129 | 225 | 185 | 191 | 162 |
| MA3 | 4 | 105 | 159 | 247 | 214 | 195 | 185 |
| MA30 | 4 | 105 | 143 | 247 | 185 | 185 | 162 |
| MA30 | 4 | 109 | 143 | 250 | 214 | 201 | 173 |

| MA32 | 4 | 105 | 131 | 244 | 221 | 185 | 166 |
| --- | --- | --- | --- | --- | --- | --- | --- |
| MA32 | 4 | 105 | 143 | 247 | 221 | 191 | 177 |
| MA4 | 4 | 105 | 131 | 225 | 203 | 185 | 162 |
| MA4 | 4 | 105 | 159 | 234 | 217 | 185 | 177 |
| MA5 | 4 | 105 | 143 | 244 | 207 | 197 | 166 |
| MA5 | 4 | 105 | 143 | 250 | 221 | 197 | 168 |
| MA6 | 4 | 105 | 131 | 254 | 214 | 195 | 166 |
| MA6 | 4 | 105 | 143 | 278 | 221 | 195 | 171 |
| MA7 | 4 | 105 | 147 | 244 | 207 | 195 | 162 |
| MA7 | 4 | 105 | 159 | 254 | 217 | 197 | 179 |
| MA8 | 4 | 96 | 159 | 240 | 214 | 185 | 166 |
| MA8 | 4 | 105 | 159 | 294 | 217 | 191 | 183 |
| MA9 | 4 | 105 | 129 | 244 | 207 | 191 | 162 |
| MA9 | 4 | 105 | 143 | 244 | 221 | 195 | 171 |
| MB1 | 5 | 105 | 143 | 254 | 221 | 191 | 193 |
| MB1 | 5 | 105 | 143 | 294 | 254 | 191 | 198 |
| MB10 | 5 | 105 | 143 | 225 | 211 | 188 | 162 |
| MB10 | 5 | 105 | 143 | 294 | 211 | 191 | 230 |
| MB12 | 5 | 105 | 143 | 247 | 203 | 191 | 162 |
| MB12 | 5 | 105 | 143 | 258 | 211 | 191 | 162 |
| MB13 | 5 | 105 | 143 | 290 | 185 | 191 | 162 |
| MB13 | 5 | 105 | 147 | 294 | 217 | 191 | 191 |
| MB14 | 5 | 105 | 143 | 298 | 185 | 191 | 162 |
| MB14 | 5 | 105 | 143 | 298 | 214 | 195 | 162 |
| MB15 | 5 | 105 | 143 | 294 | 185 | 191 | 162 |
| MB15 | 5 | 105 | 143 | 294 | 185 | 191 | 162 |
| MB16 | 5 | 105 | 143 | 247 | 217 | 191 | 155 |
| MB16 | 5 | 105 | 143 | 298 | 232 | 191 | 173 |
| MB17 | 5 | 103 | 143 | 244 | 185 | 191 | 162 |
| MB17 | 5 | 105 | 159 | 294 | 214 | 191 | 222 |
| MB18 | 5 | 105 | 143 | 244 | 211 | 191 | 162 |
| MB18 | 5 | 105 | 143 | 264 | 248 | 191 | 162 |
| MB2 | 5 | 105 | 143 | 240 | 185 | 191 | 179 |
| MB2 | 5 | 105 | 143 | 284 | 228 | 191 | 183 |
| MB20 | 5 | 105 | 143 | 247 | 207 | 191 | 155 |
| MB20 | 5 | 109 | 143 | 254 | 248 | 197 | 162 |
| MB21 | 5 | 105 | 143 | 247 | 185 | 191 | 162 |
| MB21 | 5 | 105 | 145 | 254 | 236 | 191 | 162 |
| MB22 | 5 | 105 | 143 | 244 | 185 | 191 | 162 |
| MB22 | 5 | 105 | 143 | 294 | 185 | 191 | 179 |
| MB23 | 5 | 105 | 143 | 244 | 195 | 191 | 155 |
| MB23 | 5 | 105 | 143 | 294 | 232 | 191 | 162 |
| MB24 | 5 | 105 | 143 | 244 | 180 | 191 | 162 |
| MB24 | 5 | 105 | 147 | 271 | 185 | 191 | 185 |
| MB25 | 5 | 105 | 143 | 230 | 185 | 191 | 162 |

| MB26 | 5 | 105 | 143 | 254 | 207 | 191 | 155 |
| --- | --- | --- | --- | --- | --- | --- | --- |
| MB26 | 5 | 105 | 147 | 290 | 248 | 191 | 162 |
| MB27 | 5 | 105 | 143 | 225 | 180 | 191 | 162 |
| MB27 | 5 | 105 | 143 | 254 | 214 | 191 | 193 |
| MB28 | 5 | 105 | 143 | 225 | 180 | 191 | 162 |
| MB28 | 5 | 105 | 143 | 294 | 214 | 191 | 179 |
| MB29 | 5 | 105 | 143 | 234 | 207 | 191 | 162 |
| MB29 | 5 | 105 | 149 | 247 | 207 | 191 | 183 |
| MB3 | 5 | 105 | 143 | 247 | 185 | 191 | 155 |
| MB3 | 5 | 105 | 143 | 254 | 211 | 191 | 162 |
| MB30 | 5 | 105 | 143 | 244 | 180 | 191 | 155 |
| MB30 | 5 | 105 | 143 | 360 | 232 | 191 | 179 |
| MB31 | 5 | 105 | 143 | 313 | 221 | 188 | 162 |
| MB31 | 5 | 105 | 143 | 330 | 277 | 188 | 162 |
| MB32 | 5 | 105 | 143 | 240 | 185 | 188 | 162 |
| MB32 | 5 | 105 | 143 | 360 | 244 | 191 | 185 |
| MB4 | 5 | 105 | 143 | 294 | 214 | 191 | 162 |
| MB4 | 5 | 105 | 143 | 294 | 221 | 191 | 162 |
| MB5 | 5 | 105 | 143 | 234 | 185 | 191 | 162 |
| MB5 | 5 | 105 | 143 | 247 | 207 | 191 | 171 |
| MB6 | 5 | 105 | 143 | 247 | 211 | 191 | 162 |
| MB6 | 5 | 105 | 143 | 290 | 232 | 195 | 162 |
| MB7 | 5 | 105 | 143 | 244 | 180 | 191 | 162 |
| MB7 | 5 | 105 | 143 | 254 | 185 | 195 | 162 |
| MB8 | 5 | 105 | 143 | 234 | 185 | 191 | 162 |
| MB8 | 5 | 105 | 147 | 244 | 200 | 191 | 164 |
| MB9 | 5 | 105 | 143 | 244 | 185 | 191 | 162 |
| MB9 | 5 | 105 | 147 | 244 | 214 | 191 | 162 |
| MC1 | 6 | 105 | 143 | 247 | 195 | 191 | 162 |
| MC1 | 6 | 105 | 143 | 298 | 214 | 191 | 175 |
| MC10 | 6 | 105 | 143 | 247 | 211 | 191 | 162 |
| MC10 | 6 | 105 | 147 | 290 | 232 | 191 | 173 |
| MC11 | 6 | 105 | 143 | 247 | 211 | 191 | 162 |
| MC11 | 6 | 105 | 143 | 298 | 224 | 195 | 198 |
| MC12 | 6 | 105 | 143 | 234 | 214 | 188 | 162 |
| MC12 | 6 | 105 | 155 | 244 | 217 | 191 | 162 |
| MC13 | 6 | 105 | 143 | 244 | 180 | 191 | 162 |
| MC13 | 6 | 109 | 143 | 261 | 214 | 197 | 162 |
| MC14 | 6 | 105 | 143 | 244 | 207 | 191 | 162 |
| MC14 | 6 | 105 | 147 | 294 | 217 | 195 | 162 |
| MC15 | 6 | 105 | 143 | 234 | 207 | 191 | 162 |
| MC15 | 6 | 109 | 147 | 244 | 211 | 195 | 166 |
| MC16 | 6 | 105 | 143 | 244 | 240 | 191 | 162 |
| MC16 | 6 | 105 | 143 | 264 | 240 | 191 | 171 |
| MC18 | 6 | 105 | 143 | 230 | 203 | 191 | 162 |
| MC18 | 6 | 105 | 143 | 284 | 214 | 191 | 162 |

| MC19 6 | 105 | 143 | 264 | 217 | 191 | 162 |
| --- | --- | --- | --- | --- | --- | --- |
| MC19 6 | 105 | 143 | 290 | 236 | 191 | 166 |
| MC2 6 | 105 | 143 | 234 | 207 | 191 | 162 |
| MC2 6 | 105 | 143 | 247 | 214 | 191 | 162 |
| MC20 6 | 105 | 143 | 225 | 207 | 191 | 162 |
| MC20 6 | 105 | 143 | 225 | 224 | 191 | 198 |
| MC21 6 | 105 | 143 | 264 | 214 | 191 | 162 |
| MC21 6 | 105 | 147 | 268 | 248 | 191 | 171 |
| MC22 6 | 105 | 143 | 254 | 203 | 191 | 146 |
| MC22 6 | 105 | 143 | 360 | 203 | 191 | 162 |
| MC23 6 | 105 | 143 | 254 | 200 | 191 | 162 |
| MC23 6 | 105 | 143 | 290 | 203 | 191 | 205 |
| MC24 6 | 105 | 143 | 264 | 195 | 191 | 162 |
| MC24 6 | 105 | 143 | 294 | 221 | 191 | 175 |
| MC25 6 | 105 | 129 | 234 | 217 | 191 | 162 |
| MC25 6 | 105 | 147 | 264 | 228 | 191 | 173 |
| MC26 6 | 105 | 129 | 244 | 207 | 191 | 162 |
| MC26 6 | 105 | 147 | 290 | 214 | 191 | 162 |
| MC27 6 | 105 | 143 | 225 | 211 | 185 | 166 |
| MC27 6 | 105 | 147 | 264 | 217 | 191 | 183 |
| MC28 6 | 105 | 143 | 225 | 185 | 195 | 162 |
| MC28 6 | 105 | 143 | 244 | 232 | 201 | 162 |
| MC29 6 | 105 | 143 | 225 | 207 | 191 | 162 |
| MC29 6 | 105 | 155 | 247 | 228 | 191 | 162 |
| MC3 6 | 96 | 147 | 225 | 217 | 191 | 162 |
| MC3 6 | 105 | 155 | 268 | 217 | 191 | 164 |
| MC30 6 | 105 | 143 | 244 | 211 | 191 | 162 |
| MC30 6 | 105 | 155 | 294 | 211 | 191 | 191 |
| MC31 6 | 105 | 143 | 290 | 214 | 191 | 162 |
| MC31 6 | 105 | 143 | 294 | 214 | 191 | 162 |
| MC32 6 | 105 | 143 | 247 | 207 | 191 | 162 |
| MC32 6 | 105 | 143 | 290 | 211 | 195 | 173 |
| MC4 6 | 105 | 143 | 247 | 228 | 191 | 162 |
| MC4 6 | 105 | 147 | 290 | 232 | 197 | 162 |
| MC5 6 | 105 | 143 | 261 | 207 | 191 | 162 |
| MC5 6 | 105 | 143 | 290 | 214 | 191 | 162 |
| MC6 6 | 105 | 143 | 247 | 207 | 191 | 162 |
| MC6 6 | 105 | 143 | 261 | 214 | 191 | 168 |
| MC7 6 | 105 | 155 | 261 | 217 | 191 | 162 |
| MC7 6 | 105 | 155 | 290 | 224 | 195 | 162 |
| MC8 6 | 105 | 143 | 234 | 214 | 185 | 162 |
| MC8 6 | 105 | 147 | 261 | 217 | 191 | 162 |
| MC9 6 | 105 | 143 | 294 | 195 | 191 | 162 |
| MC9 6 | 105 | 143 | 313 | 211 | 191 | 162 |

| AA10 | 7 | 105 | 143 | 225 | 185 | 191 | 173 |
| --- | --- | --- | --- | --- | --- | --- | --- |
| AA10 | 7 | 105 | 147 | 247 | 207 | 191 | 191 |
| AA11 | 7 | 105 | 143 | 237 | 211 | 191 | 185 |
| AA11 | 7 | 107 | 147 | 254 | 211 | 191 | 198 |
| AA12 | 7 | 105 | 129 | 244 | 221 | 191 | 162 |
| AA12 | 7 | 105 | 147 | 254 | 221 | 191 | 162 |
| AA13 | 7 | 105 | 143 | 247 | 232 | 191 | 162 |
| AA13 | 7 | 105 | 147 | 258 | 261 | 191 | 162 |
| AA14 | 7 | 105 | 143 | 225 | 217 | 188 | 162 |
| AA14 | 7 | 105 | 147 | 298 | 221 | 191 | 183 |
| AA15 | 7 | 105 | 143 | 225 | 217 | 191 | 162 |
| AA15 | 7 | 105 | 151 | 290 | 224 | 191 | 198 |
| AA16 | 7 | 105 | 143 | 247 | 214 | 191 | 162 |
| AA16 | 7 | 105 | 143 | 250 | 232 | 197 | 222 |
| AA17 | 7 | 105 | 129 | 230 | 214 | 191 | 162 |
| AA17 | 7 | 105 | 147 | 298 | 214 | 191 | 222 |
| AA18 | 7 | 105 | 143 | 230 | 214 | 191 | 162 |
| AA18 | 7 | 105 | 143 | 244 | 217 | 191 | 205 |
| AA19 | 7 | 105 | 143 | 225 | 211 | 191 | 162 |
| AA19 | 7 | 105 | 143 | 225 | 211 | 191 | 198 |
| AA2 | 7 | 105 | 143 | 225 | 214 | 191 | 185 |
| AA2 | 7 | 105 | 143 | 250 | 217 | 191 | 198 |
| AA20 | 7 | 105 | 129 | 225 | 211 | 188 | 183 |
| AA20 | 7 | 105 | 147 | 298 | 236 | 191 | 222 |
| AA21 | 7 | 105 | 143 | 244 | 211 | 191 | 158 |
| AA21 | 7 | 105 | 143 | 258 | 214 | 191 | 158 |
| AA22 | 7 | 105 | 129 | 225 | 207 | 191 | 191 |
| AA22 | 7 | 105 | 147 | 230 | 224 | 191 | 205 |
| AA23 | 7 | 105 | 143 | 258 | 221 | 191 | 175 |
| AA23 | 7 | 107 | 147 | 298 | 221 | 191 | 179 |
| AA24 | 7 | 105 | 143 | 230 | 211 | 191 | 158 |
| AA24 | 7 | 105 | 143 | 247 | 214 | 191 | 162 |
| AA25 | 7 | 105 | 143 | 244 | 211 | 191 | 198 |
| AA25 | 7 | 105 | 143 | 244 | 211 | 191 | 222 |
| AA26 | 7 | 105 | 143 | 244 | 214 | 191 | 162 |
| AA26 | 7 | 105 | 147 | 244 | 217 | 195 | 205 |
| AA27 | 7 | 105 | 143 | 258 | 221 | 191 | 179 |
| AA27 | 7 | 107 | 143 | 298 | 221 | 191 | 205 |
| AA28 | 7 | 105 | 143 | 244 | 236 | 191 | 191 |
| AA28 | 7 | 105 | 143 | 298 | 240 | 191 | 198 |
| AA29 | 7 | 105 | 129 | 244 | 217 | 191 | 185 |
| AA29 | 7 | 105 | 129 | 244 | 224 | 191 | 191 |
| AA3 | 7 | 105 | 143 | 225 | 200 | 191 | 183 |
| AA3 | 7 | 107 | 143 | 237 | 221 | 191 | 185 |

| AA31 | 7 | 105 | 143 | 230 | 217 | 191 | 162 |
| --- | --- | --- | --- | --- | --- | --- | --- |
| AA31 | 7 | 105 | 147 | 258 | 228 | 191 | 205 |
| AA32 | 7 | 105 | 129 | 244 | 211 | 191 | 191 |
| AA32 | 7 | 105 | 143 | 244 | 211 | 201 | 193 |
| AA4 | 7 | 105 | 143 | 247 | 228 | 191 | 162 |
| AA4 | 7 | 107 | 143 | 294 | 228 | 195 | 173 |
| AA5 | 7 | 105 | 143 | 225 | 211 | 191 | 162 |
| AA5 | 7 | 107 | 147 | 247 | 232 | 191 | 183 |
| AA6 | 7 | 105 | 143 | 225 | 207 | 191 | 193 |
| AA6 | 7 | 105 | 143 | 230 | 224 | 191 | 205 |
| AA7 | 7 | 105 | 143 | 244 | 214 | 191 | 198 |
| AA7 | 7 | 105 | 147 | 298 | 214 | 191 | 222 |
| AA8 | 7 | 105 | 143 | 244 | 214 | 191 | 185 |
| AA8 | 7 | 105 | 143 | 298 | 217 | 191 | 205 |
| AA9 | 7 | 105 | 143 | 247 | 211 | 191 | 179 |
| AA9 | 7 | 105 | 143 | 247 | 217 | 195 | 185 |
| AB1 | 8 | 105 | 143 | 250 | 214 | 197 | 162 |
| AB1 | 8 | 105 | 143 | 261 | 240 | 197 | 185 |
| AB10 | 8 | 105 | 143 | 234 | 211 | 191 | 183 |
| AB10 | 8 | 105 | 145 | 302 | 228 | 191 | 185 |
| AB11 | 8 | 105 | 143 | 244 | 211 | 191 | 179 |
| AB11 | 8 | 105 | 143 | 244 | 211 | 197 | 181 |
| AB12 | 8 | 105 | 143 | 261 | 211 | 188 | 162 |
| AB12 | 8 | 105 | 143 | 302 | 244 | 191 | 185 |
| AB13 | 8 | 105 | 143 | 261 | 214 | 191 | 162 |
| AB13 | 8 | 105 | 143 | 268 | 244 | 197 | 185 |
| AB14 | 8 | 105 | 143 | 234 | 185 | 188 | 164 |
| AB14 | 8 | 105 | 143 | 261 | 217 | 188 | 173 |
| AB15 | 8 | 105 | 143 | 244 | 211 | 191 | 162 |
| AB15 | 8 | 105 | 145 | 302 | 236 | 191 | 162 |
| AB16 | 8 | 105 | 143 | 240 | 211 | 188 | 171 |
| AB16 | 8 | 105 | 143 | 258 | 240 | 191 | 179 |
| AB17 | 8 | 105 | 143 | 234 | 211 | 191 | 181 |
| AB17 | 8 | 105 | 143 | 302 | 211 | 191 | 183 |
| AB18 | 8 | 105 | 143 | 244 | 228 | 191 | 164 |
| AB18 | 8 | 105 | 143 | 247 | 236 | 191 | 173 |
| AB19 | 8 | 105 | 143 | 250 | 211 | 191 | 162 |
| AB19 | 8 | 105 | 143 | 290 | 211 | 191 | 185 |
| AB2 | 8 | 99 | 143 | 244 | 211 | 188 | 164 |
| AB2 | 8 | 105 | 159 | 244 | 211 | 191 | 177 |
| AB20 | 8 | 105 | 143 | 237 | 228 | 191 | 171 |
| AB20 | 8 | 105 | 143 | 290 | 244 | 191 | 191 |
| AB21 | 8 | 99 | 143 | 230 | 211 | 191 | 162 |
| AB21 | 8 | 105 | 143 | 298 | 211 | 191 | 185 |
| AB22 | 8 | 105 | 143 | 247 | 211 | 191 | 162 |

| AB23 | 8 | 105 | 143 | 298 | 211 | 188 | 162 |
| --- | --- | --- | --- | --- | --- | --- | --- |
| AB23 | 8 | 105 | 143 | 302 | 211 | 191 | 185 |
| AB24 | 8 | 105 | 143 | 240 | 211 | 191 | 162 |
| AB24 | 8 | 105 | 143 | 261 | 217 | 197 | 164 |
| AB25 | 8 | 105 | 143 | 244 | 211 | 191 | 158 |
| AB25 | 8 | 105 | 143 | 298 | 211 | 201 | 162 |
| AB26 | 8 | 105 | 143 | 237 | 211 | 188 | 185 |
| AB26 | 8 | 105 | 143 | 298 | 211 | 191 | 185 |
| AB27 | 8 | 105 | 111 | 237 | 211 | 188 | 162 |
| AB27 | 8 | 105 | 143 | 247 | 214 | 191 | 181 |
| AB28 | 8 | 105 | 129 | 244 | 217 | 188 | 162 |
| AB28 | 8 | 105 | 143 | 244 | 217 | 188 | 185 |
| AB29 | 8 | 105 | 143 | 234 | 207 | 191 | 162 |
| AB29 | 8 | 105 | 143 | 298 | 269 | 191 | 164 |
| AB3 | 8 | 105 | 143 | 230 | 211 | 188 | 162 |
| AB3 | 8 | 105 | 143 | 237 | 211 | 191 | 181 |
| AB30 | 8 | 105 | 143 | 258 | 269 | 191 | 164 |
| AB30 | 8 | 105 | 143 | 268 | 269 | 191 | 164 |
| AB31 | 8 | 105 | 143 | 218 | 191 | 191 | 162 |
| AB31 | 8 | 105 | 143 | 298 | 211 | 191 | 164 |
| AB32 | 8 | 105 | 143 | 230 | 207 | 188 | 162 |
| AB32 | 8 | 105 | 143 | 244 | 217 | 191 | 185 |
| AB4 | 8 | 105 | 143 | 230 | 214 | 191 | 162 |
| AB4 | 8 | 105 | 143 | 230 | 248 | 191 | 162 |
| AB5 | 8 | 105 | 143 | 244 | 211 | 191 | 164 |
| AB5 | 8 | 105 | 143 | 250 | 217 | 191 | 181 |
| AB6 | 8 | 105 | 143 | 261 | 211 | 188 | 162 |
| AB6 | 8 | 105 | 143 | 268 | 211 | 191 | 198 |
| AB7 | 8 | 99 | 143 | 230 | 211 | 191 | 162 |
| AB7 | 8 | 105 | 143 | 250 | 214 | 191 | 185 |
| AB8 | 8 | 105 | 143 | 244 | 214 | 191 | 162 |
| AB8 | 8 | 105 | 143 | 268 | 240 | 197 | 185 |
| AB9 | 8 | 99 | 143 | 234 | 214 | 188 | 185 |
| AB9 | 8 | 105 | 143 | 244 | 214 | 191 | 185 |
| AC1 | 9 | 105 | 143 | 237 | 217 | 191 | 158 |

| AC15 | 9 | 105 | 141 | 237 | 221 | 191 | 171 |
| --- | --- | --- | --- | --- | --- | --- | --- |
| AC15 | 9 | 105 | 143 | 313 | 261 | 201 | 173 |
| AC16 | 9 | 99 | 155 | 237 | 211 | 191 | 162 |
| AC16 | 9 | 105 | 157 | 268 | 214 | 191 | 175 |
| AC17 | 9 | 105 | 151 | 230 | 180 | 193 | 171 |
| AC17 | 9 | 105 | 155 | 271 | 211 | 193 | 183 |
| AC19 | 9 | 105 | 129 | 230 | 217 | 191 | 162 |
| AC19 | 9 | 105 | 155 | 234 | 228 | 197 | 181 |
| AC2 | 9 | 105 | 151 | 237 | 211 | 191 | 162 |
| AC2 | 9 | 105 | 155 | 271 | 217 | 193 | 183 |
| AC20 | 9 | 105 | 155 | 230 | 207 | 191 | 171 |
| AC20 | 9 | 111 | 157 | 264 | 214 | 201 | 191 |
| AC21 | 9 | 105 | 143 | 230 | 211 | 191 | 162 |
| AC21 | 9 | 105 | 143 | 250 | 211 | 191 | 164 |
| AC22 | 9 | 105 | 157 | 230 | 211 | 191 | 162 |
| AC22 | 9 | 105 | 157 | 244 | 236 | 201 | 193 |
| AC23 | 9 | 105 | 143 | 244 | 217 | 191 | 162 |
| AC23 | 9 | 105 | 143 | 247 | 236 | 195 | 162 |
| AC24 | 9 | 111 | 155 | 237 | 191 | 191 | 162 |
| AC24 | 9 | 111 | 155 | 244 | 214 | 191 | 179 |
| AC25 | 9 | 105 | 129 | 234 | 214 | 197 | 162 |
| AC25 | 9 | 105 | 157 | 271 | 244 | 197 | 185 |
| AC26 | 9 | 105 | 143 | 234 | 203 | 191 | 162 |
| AC26 | 9 | 105 | 143 | 247 | 224 | 197 | 162 |
| AC27 | 9 | 105 | 143 | 230 | 214 | 191 | 158 |
| AC27 | 9 | 105 | 143 | 244 | 228 | 191 | 162 |
| AC28 | 9 | 105 | 143 | 244 | 207 | 191 | 171 |
| AC28 | 9 | 105 | 157 | 258 | 214 | 191 | 171 |
| AC29 | 9 | 105 | 155 | 230 | 214 | 191 | 162 |
| AC29 | 9 | 105 | 155 | 254 | 228 | 191 | 171 |
| AC3 | 9 | 105 | 143 | 247 | 214 | 191 | 162 |
| AC3 | 9 | 105 | 143 | 258 | 240 | 191 | 162 |
| AC30 | 9 | 105 | 141 | 250 | 211 | 191 | 162 |
| AC30 | 9 | 105 | 143 | 261 | 254 | 191 | 164 |
| AC31 | 9 | 105 | 141 | 237 | 180 | 191 | 162 |

| AC8 | 9 | 105 | 141 | 230 | 207 | 191 | 162 |  |
| --- | --- | --- | --- | --- | --- | --- | --- | --- |
| AC8 | 9 | 105 | 155 | 234 | 228 | 197 | 171 |  |
| AC9 | 9 | 105 | 143 | 234 | 217 | 191 | 162 |  |
| AC9 | 9 | 105 | 155 | 254 | 217 | 193 | 171 |  |
| ES963 |  | 10 | 107 | 149 | 230 | 191 | 193 | 155 |
| ES963 |  | 10 | 111 | 153 | 298 | 191 | 193 | 158 |
| ES964 |  | 10 | 105 | 133 | 268 | 185 | 188 | 162 |
| ES964 |  | 10 | 105 | 143 | 268 | 244 | 203 | 183 |
| ES965 |  | 10 | 99 | 133 | 230 | 248 | 193 | 162 |
| ES965 |  | 10 | 105 | 157 | 230 | 265 | 195 | 171 |
| ES966 |  | 10 | 105 | 133 | 230 | 211 | 191 | 158 |
| ES966 |  | 10 | 111 | 145 | 250 | 232 | 195 | 183 |
| ES967 |  | 10 | 99 | 123 | 247 | 185 | 193 | 158 |
| ES967 |  | 10 | 111 | 147 | 268 | 191 | 195 | 173 |
| ES969 |  | 10 | 99 | 133 | 250 | 244 | 195 | 158 |
| ES969 |  | 10 | 111 | 133 | 250 | 298 | 195 | 205 |
| ES970 |  | 10 | 107 | 133 | 230 | 191 | 188 | 162 |
| ES970 |  | 10 | 111 | 143 | 250 | 217 | 191 | 162 |
| ES973 |  | 10 | 107 | 133 | 225 | 217 | 195 | 158 |
| ES973 |  | 10 | 111 | 153 | 268 | 298 | 197 | 158 |
| ES974 |  | 10 | 107 | 133 | 247 | 185 | 188 | 158 |
| ES974 |  | 10 | 107 | 145 | 268 | 265 | 188 | 158 |
| ES975 |  | 10 | 99 | 133 | 230 | 191 | 191 | 162 |
| ES975 |  | 10 | 107 | 149 | 230 | 265 | 195 | 205 |
| ES976 |  | 10 | 107 | 133 | 223 | 236 | 191 | 155 |
| ES976 |  | 10 | 107 | 153 | 268 | 265 | 193 | 158 |
| ES977 |  | 10 | 99 | 123 | 268 | 211 | 188 | 171 |
| ES977 |  | 10 | 105 | 155 | 268 | 217 | 193 | 173 |
| ES978 |  | 10 | 99 | 143 | 230 | 185 | 188 | 158 |
| ES978 |  | 10 | 107 | 149 | 268 | 211 | 193 | 183 |
| ES979 |  | 10 | 99 | 143 | 230 | 211 | 191 | 171 |
| ES979 |  | 10 | 107 | 145 | 268 | 217 | 193 | 205 |
| ES980 |  | 10 | 105 | 153 | 250 | 191 | 188 | 158 |
| ES980 |  | 10 | 107 | 155 | 268 | 244 | 188 | 162 |
| ES981 |  | 10 | 107 | 143 | 247 | 195 | 191 | 158 |
| ES981 |  | 10 | 111 | 155 | 271 | 217 | 191 | 183 |

| ES987 |  | 10 | 107 | 149 | 225 | 211 | 201 | 158 |
| --- | --- | --- | --- | --- | --- | --- | --- | --- |
| ES987 |  | 10 | 111 | 153 | 268 | 211 | 203 | 205 |
| ES988 |  | 10 | 107 | 143 | 247 | 185 | 191 | 162 |
| ES988 |  | 10 | 111 | 145 | 268 | 265 | 191 | 173 |
| ES989 |  | 10 | 105 | 145 | 230 | 185 | 193 | 155 |
| ES989 |  | 10 | 111 | 153 | 298 | 191 | 193 | 173 |
| ES990 |  | 10 | 99 | 143 | 247 | 185 | 193 | 158 |
| ES990 |  | 10 | 99 | 143 | 250 | 191 | 193 | 173 |
| ES991 |  | 10 | 107 | 143 | 247 | 191 | 193 | 173 |
| ES991 |  | 10 | 111 | 149 | 250 | 228 | 203 | 173 |
| ES992 |  | 10 | 111 | 155 | 225 | 236 | 191 | 162 |
| ES992 |  | 10 | 111 | 155 | 298 | 265 | 193 | 173 |
| ES993 |  | 10 | 105 | 143 | 250 | 191 | 191 | 158 |
| ES993 |  | 10 | 107 | 143 | 268 | 217 | 191 | 158 |
| ES994 |  | 10 | 105 | 133 | 223 | 191 | 188 | 158 |
| ES994 |  | 10 | 111 | 143 | 230 | 298 | 193 | 162 |
| ES995 |  | 10 | 99 | 133 | 223 | 211 | 191 | 158 |
| ES995 |  | 10 | 107 | 145 | 268 | 298 | 191 | 158 |
| AD1 | 11 | 105 | 133 | 250 | 191 | 195 | 155 |  |
| AD1 | 11 | 111 | 143 | 268 | 248 | 195 | 162 |  |
| AD10 | 11 | 105 | 143 | 230 | 191 | 191 | 162 |  |
| AD10 | 11 | 105 | 143 | 230 | 265 | 197 | 162 |  |
| AD11 | 11 | 99 | 143 | 230 | 211 | 191 | 158 |  |
| AD11 | 11 | 99 | 155 | 250 | 244 | 195 | 158 |  |
| AD12 | 11 | 107 | 133 | 230 | 191 | 191 | 158 |  |
| AD12 | 11 | 111 | 133 | 230 | 236 | 191 | 183 |  |
| AD13 | 11 | 111 | 133 | 250 | 211 | 191 | 158 |  |
| AD13 | 11 | 111 | 143 | 298 | 265 | 191 | 183 |  |
| AD14 | 11 | 99 | 133 | 225 | 191 | 191 | 155 |  |
| AD14 | 11 | 99 | 143 | 230 | 244 | 195 | 162 |  |
| AD15 | 11 | 105 | 145 | 250 | 185 | 191 | 158 |  |
| AD15 | 11 | 107 | 157 | 298 | 244 | 203 | 171 |  |
| AD2 | 11 | 99 | 143 | 225 | 185 | 191 | 155 |  |
| AD2 | 11 | 111 | 145 | 250 | 240 | 191 | 162 |  |
| AD3 | 11 | 99 | 133 | 230 | 185 | 188 | 158 |  |
| AD3 | 11 | 111 | 143 | 230 | 211 | 195 | 162 |  |
| AD4 | 11 | 107 | 133 | 244 | 191 | 191 | 183 |  |

| HL92812 | 103 | 143 | 271 | 185 | 188 | 168 |
| --- | --- | --- | --- | --- | --- | --- |
| HL92812 | 103 | 145 | 271 | 240 | 197 | 177 |
| HL93012 | 103 | 139 | 237 | 214 | 195 | 171 |
| HL93012 | 105 | 145 | 264 | 240 | 197 | 179 |
| HL93112 | 105 | 147 | 250 | 200 | 191 | 166 |
| HL93112 | 107 | 147 | 264 | 232 | 195 | 168 |
| HL93312 | 103 | 143 | 244 | 232 | 188 | 160 |
| HL93312 | 105 | 147 | 271 | 240 | 197 | 168 |
| HL93412 | 103 | 145 | 240 | 221 | 188 | 171 |
| HL93412 | 105 | 145 | 271 | 232 | 195 | 177 |
| HL93512 | 107 | 139 | 247 | 240 | 191 | 160 |
| HL93512 | 107 | 147 | 254 | 240 | 191 | 164 |
| HL93612 | 105 | 139 | 223 | 185 | 191 | 158 |
| HL93612 | 105 | 145 | 250 | 217 | 193 | 177 |
| HL93712 | 103 | 147 | 234 | 185 | 191 | 160 |
| HL93712 | 105 | 165 | 244 | 185 | 191 | 177 |
| HL93812 | 99 | 139 | 223 | 200 | 195 | 168 |
| HL93812 | 107 | 143 | 223 | 274 | 201 | 168 |
| HL93912 | 99 | 147 | 237 | 185 | 195 | 166 |
| HL93912 | 107 | 155 | 254 | 214 | 195 | 168 |
| HL94012 | 105 | 139 | 223 | 214 | 188 | 158 |
| HL94012 | 107 | 147 | 240 | 240 | 191 | 164 |
| HL94112 | 105 | 133 | 237 | 214 | 197 | 164 |
| HL94112 | 105 | 143 | 271 | 232 | 197 | 164 |
| HL94212 | 103 | 139 | 244 | 214 | 197 | 179 |
| HL94212 | 105 | 171 | 254 | 232 | 197 | 179 |
| HL94312 | 105 | 133 | 234 | 248 | 191 | 164 |
| HL94312 | 105 | 139 | 271 | 261 | 203 | 168 |
| HL94412 | 103 | 143 | 250 | 261 | 197 | 158 |
| HL94412 | 105 | 143 | 264 | 288 | 197 | 164 |
| HL94512 | 103 | 143 | 237 | 240 | 191 | 168 |
| HL94512 | 103 | 147 | 271 | 240 | 195 | 171 |
| HL94612 | 105 | 139 | 237 | 240 | 197 | 171 |
| HL94612 | 107 | 157 | 271 | 240 | 197 | 177 |
| HL94712 | 105 | 139 | 250 | 232 | 191 | 158 |
| HL94712 | 107 | 153 | 271 | 248 | 195 | 177 |
| HL94812 | 105 | 137 | 234 | 261 | 193 | 160 |
| HL94812 | 107 | 143 | 271 | 274 | 193 | 179 |

| HL95512 | 105 | 139 | 254 | 214 | 195 | 158 |  |
| --- | --- | --- | --- | --- | --- | --- | --- |
| HL95512 | 107 | 139 | 271 | 232 | 195 | 166 |  |
| HL95712 | 105 | 139 | 230 | 232 | 191 | 164 |  |
| HL95712 | 105 | 165 | 258 | 236 | 195 | 168 |  |
| HL95812 | 105 | 165 | 244 | 236 | 191 | 158 |  |
| HL95812 | 105 | 171 | 271 | 274 | 201 | 158 |  |
| HL95912 | 103 | 145 | 250 | 228 | 188 | 155 |  |
| HL95912 | 105 | 145 | 271 | 248 | 201 | 177 |  |
| HL96012 | 107 | 139 | 234 | 185 | 191 | 164 |  |
| HL96012 | 107 | 139 | 250 | 232 | 191 | 166 |  |
| HL96112 | 103 | 143 | 234 | 236 | 191 | 166 |  |
| HL96112 | 105 | 165 | 234 | 248 | 195 | 166 |  |
| HL96212 | 103 | 143 | 237 | 232 | 191 | 160 |  |
| HL96212 | 105 | 147 | 274 | 240 | 197 | 171 |  |
| KL1001 | 13 | 99 | 143 | 244 | 240 | 188 | 168 |
| KL1001 | 13 | 105 | 155 | 250 | 248 | 195 | 171 |
| KL1004 | 13 | 103 | 143 | 237 | 191 | 188 | 168 |
| KL1004 | 13 | 105 | 143 | 250 | 248 | 188 | 181 |
| KL1005 | 13 | 99 | 143 | 234 | 236 | 188 | 171 |
| KL1005 | 13 | 107 | 153 | 240 | 236 | 193 | 171 |
| KL1006 | 13 | 103 | 143 | 240 | 185 | 188 | 155 |
| KL1006 | 13 | 105 | 143 | 261 | 185 | 193 | 168 |
| KL1007 | 13 | 99 | 143 | 247 | 240 | 193 | 146 |
| KL1007 | 13 | 105 | 147 | 274 | 240 | 193 | 171 |
| KL1008 | 13 | 107 | 143 | 234 | 191 | 195 | 171 |
| KL1008 | 13 | 107 | 143 | 250 | 265 | 195 | 175 |
| KL1010 | 13 | 99 | 143 | 247 | 191 | 188 | 155 |
| KL1010 | 13 | 99 | 143 | 247 | 254 | 188 | 168 |
| KL1011 | 13 | 105 | 143 | 250 | 200 | 195 | 155 |
| KL1011 | 13 | 107 | 155 | 250 | 228 | 201 | 171 |
| KL1012 | 13 | 99 | 135 | 234 | 240 | 188 | 158 |
| KL1012 | 13 | 107 | 151 | 254 | 240 | 197 | 171 |
| KL1014 | 13 | 105 | 143 | 240 | 240 | 191 | 171 |
| KL1014 | 13 | 105 | 143 | 250 | 248 | 193 | 181 |
| KL1016 | 13 | 99 | 143 | 261 | 240 | 195 | 146 |
| KL1016 | 13 | 103 | 143 | 274 | 254 | 201 | 171 |
| KL1017 | 13 | 99 | 135 | 234 | 191 | 191 | 168 |
| KL1017 | 13 | 99 | 135 | 250 | 240 | 193 | 168 |
| KL1018 | 13 | 99 | 143 | 234 | 228 | 203 | 160 |

| KL1022 | 13 | 99 | 135 | 247 | 240 | 195 | 155 |
| --- | --- | --- | --- | --- | --- | --- | --- |
| KL1022 | 13 | 107 | 143 | 258 | 313 | 201 | 168 |
| KL1023 | 13 | 105 | 143 | 234 | 185 | 195 | 146 |
| KL1023 | 13 | 107 | 143 | 261 | 240 | 195 | 171 |
| KL1024 | 13 | 105 | 143 | 250 | 248 | 193 | 155 |
| KL1024 | 13 | 107 | 155 | 274 | 248 | 197 | 158 |
| KL1025 | 13 | 105 | 135 | 237 | 240 | 193 | 155 |
| KL1025 | 13 | 107 | 143 | 250 | 240 | 193 | 168 |
| KL1028 | 13 | 99 | 143 | 244 | 240 | 188 | 158 |
| KL1028 | 13 | 107 | 143 | 274 | 244 | 201 | 181 |
| KL1030 | 13 | 105 | 143 | 237 | 240 | 193 | 158 |
| KL1030 | 13 | 107 | 147 | 250 | 254 | 195 | 181 |
| KL1031 | 13 | 99 | 143 | 237 | 191 | 191 | 164 |
| KL1031 | 13 | 99 | 151 | 274 | 240 | 195 | 168 |
| KL1032 | 13 | 99 | 143 | 274 | 240 | 193 | 168 |
| KL1032 | 13 | 99 | 155 | 274 | 261 | 193 | 171 |
| KL1033 | 13 | 99 | 143 | 237 | 185 | 193 | 160 |
| KL1033 | 13 | 99 | 155 | 250 | 313 | 195 | 168 |
| KL1034 | 13 | 103 | 135 | 234 | 240 | 191 | 175 |
| KL1034 | 13 | 107 | 151 | 240 | 240 | 201 | 177 |
| KL1036 | 13 | 105 | 143 | 234 | 240 | 188 | 158 |
| KL1036 | 13 | 107 | 143 | 240 | 240 | 193 | 168 |
| KL1037 | 13 | 99 | 143 | 240 | 236 | 191 | 155 |
| KL1037 | 13 | 107 | 143 | 250 | 240 | 201 | 171 |
| KL1038 | 13 | 99 | 143 | 240 | 244 | 193 | 158 |
| KL1038 | 13 | 99 | 155 | 261 | 248 | 201 | 168 |
| KL99613 | 99 | 151 | 250 | 185 | 195 | 168 |  |
| KL99613 | 107 | 151 | 271 | 248 | 201 | 171 |  |
| KL99713 | 103 | 143 | 234 | 214 | 193 | 146 |  |
| KL99713 | 107 | 143 | 274 | 248 | 203 | 171 |  |
| KL99813 | 99 | 143 | 240 | 214 | 188 | 155 |  |
| KL99813 | 105 | 153 | 250 | 228 | 193 | 168 |  |
| FA1 14 | 105 | 123 | 250 | 195 | 191 | 151 |  |
| FA1 14 | 105 | 151 | 250 | 195 | 191 | 155 |  |
| FA10 14 | 99 | 123 | 240 | 195 | 191 | 155 |  |
| FA10 14 | 105 | 143 | 250 | 195 | 191 | 175 |  |

| FA3 14 | 105 | 123 | 230 | 191 | 191 | 151 |
| --- | --- | --- | --- | --- | --- | --- |
| FA3 14 | 105 | 147 | 247 | 217 | 191 | 155 |
| FA4 14 | 105 | 129 | 234 | 221 | 191 | 151 |
| FA4 14 | 105 | 155 | 237 | 232 | 191 | 155 |
| FA5 14 | 105 | 143 | 237 | 217 | 188 | 151 |
| FA5 14 | 105 | 147 | 274 | 217 | 191 | 155 |
| FA6 14 | 103 | 123 | 230 | 214 | 195 | 151 |
| FA6 14 | 105 | 123 | 250 | 214 | 195 | 151 |
| FA7 14 | 105 | 141 | 230 | 228 | 191 | 151 |
| FA7 14 | 105 | 145 | 247 | 248 | 191 | 155 |
| FA8 14 | 105 | 123 | 250 | 185 | 188 | 155 |
| FA8 14 | 105 | 135 | 261 | 269 | 193 | 155 |
| FA86614 | 105 | 143 | 230 | 232 | 195 | 146 |
| FA86614 | 105 | 143 | 237 | 240 | 195 | 155 |
| FA86714 | 105 | 123 | 230 | 221 | 195 | 168 |
| FA86714 | 105 | 135 | 250 | 240 | 195 | 179 |
| FA86814 | 103 | 129 | 230 | 195 | 191 | 133 |
| FA86814 | 105 | 147 | 250 | 203 | 191 | 171 |
| FA86914 | 105 | 123 | 230 | 203 | 195 | 146 |
| FA86914 | 105 | 123 | 230 | 240 | 195 | 173 |
| FA87014 | 103 | 123 | 237 | 232 | 191 | 151 |
| FA87014 | 105 | 123 | 274 | 274 | 191 | 151 |
| FA87114 | 105 | 123 | 230 | 207 | 193 | 151 |
| FA87114 | 107 | 143 | 247 | 236 | 193 | 168 |
| FA87214 | 105 | 123 | 225 | 240 | 191 | 146 |
| FA87214 | 105 | 123 | 268 | 274 | 191 | 175 |
| FA87314 | 105 | 123 | 250 | 207 | 193 | 151 |
| FA87314 | 105 | 151 | 258 | 232 | 193 | 160 |
| FA87414 | 103 | 123 | 230 | 200 | 191 | 173 |
| FA87414 | 105 | 123 | 258 | 240 | 193 | 175 |
| FA87514 | 103 | 123 | 230 | 228 | 191 | 151 |
| FA87514 | 105 | 143 | 237 | 248 | 193 | 151 |
| FA87614 | 107 | 123 | 230 | 203 | 191 | 151 |
| FA87614 | 107 | 155 | 258 | 236 | 193 | 175 |
| FA87714 | 105 | 123 | 237 | 228 | 191 | 151 |
| FA87714 | 105 | 123 | 258 | 274 | 191 | 151 |

| FA92214 | 103 | 123 | 230 | 200 | 191 | 151 |  |
| --- | --- | --- | --- | --- | --- | --- | --- |
| FA92214 | 105 | 123 | 230 | 217 | 191 | 151 |  |
| WA1.711 | 15 | 105 | 147 | 240 | 207 | 195 | 164 |
| WA1.711 | 15 | 105 | 177 | 244 | 240 | 195 | 175 |
| WA1.714 | 15 | 105 | 149 | 225 | 217 | 191 | 164 |
| WA1.714 | 15 | 105 | 155 | 264 | 254 | 195 | 208 |
| WA1.725 | 15 | 105 | 137 | 230 | 203 | 191 | 171 |
| WA1.725 | 15 | 105 | 173 | 230 | 217 | 197 | 181 |
| WA1070 | 15 | 105 | 141 | 230 | 203 | 191 | 166 |
| WA1070 | 15 | 105 | 155 | 247 | 214 | 191 | 175 |
| WA1071 | 15 | 99 | 145 | 237 | 217 | 197 | 168 |
| WA1071 | 15 | 105 | 161 | 254 | 224 | 197 | 171 |
| WA1072 | 15 | 99 | 145 | 237 | 240 | 195 | 164 |
| WA1072 | 15 | 105 | 161 | 254 | 248 | 195 | 166 |
| WA1086 | 15 | 103 | 145 | 218 | 248 | 193 | 166 |
| WA1086 | 15 | 105 | 149 | 237 | 248 | 193 | 171 |
| WA1087 | 15 | 105 | 153 | 230 | 214 | 191 | 168 |
| WA1087 | 15 | 105 | 155 | 240 | 228 | 195 | 171 |
| WA1088 | 15 | 105 | 145 | 240 | 224 | 191 | 171 |
| WA1088 | 15 | 107 | 149 | 254 | 248 | 191 | 173 |
| WA1089 | 15 | 105 | 149 | 225 | 217 | 191 | 164 |
| WA1089 | 15 | 105 | 155 | 264 | 254 | 195 | 208 |
| WA1090 | 15 | 105 | 147 | 240 | 207 | 195 | 164 |
| WA1090 | 15 | 105 | 177 | 244 | 240 | 195 | 175 |
| WA2.714 | 15 | 105 | 145 | 237 | 224 | 191 | 171 |
| WA2.714 | 15 | 107 | 149 | 254 | 248 | 191 | 173 |
| WA237 | 15 | 99 | 145 | 250 | 203 | 195 | 168 |
| WA237 | 15 | 105 | 145 | 254 | 221 | 195 | 171 |
| WA238 | 15 | 105 | 151 | 237 | 191 | 197 | 164 |
| WA238 | 15 | 105 | 173 | 250 | 228 | 197 | 198 |
| WA239 | 15 | 105 | 153 | 234 | 221 | 197 | 168 |
| WA239 | 15 | 105 | 177 | 247 | 274 | 197 | 171 |
| WA240 | 15 | 105 | 157 | 211 | 224 | 197 | 164 |
| WA240 | 15 | 105 | 177 | 264 | 228 | 203 | 171 |
| WA307 | 15 | 99 | 143 | 240 | 203 | 193 | 171 |
| WA307 | 15 | 105 | 145 | 247 | 207 | 195 | 173 |
| WA309 | 15 | 105 | 137 | 211 | 224 | 191 | 171 |

| WA552 | 15 | 99 | 139 | 247 | 203 | 195 | 164 |
| --- | --- | --- | --- | --- | --- | --- | --- |
| WA552 | 15 | 103 | 145 | 250 | 203 | 195 | 171 |
| WA553 | 15 | 105 | 153 | 223 | 254 | 193 | 177 |
| WA553 | 15 | 105 | 153 | 234 | 298 | 203 | 185 |
| WA579 | 15 | 99 | 135 | 230 | 214 | 201 | 168 |
| WA579 | 15 | 105 | 167 | 237 | 217 | 201 | 193 |
| WA592 | 15 | 103 | 147 | 250 | 203 | 197 | 173 |
| WA592 | 15 | 105 | 147 | 250 | 207 | 197 | 198 |
| WA593 | 15 | 103 | 145 | 211 | 217 | 191 | 168 |
| WA593 | 15 | 105 | 149 | 240 | 261 | 193 | 171 |
| WA598 | 15 | 99 | 145 | 240 | 207 | 197 | 164 |
| WA598 | 15 | 105 | 151 | 247 | 224 | 197 | 173 |
| WA599 | 15 | 103 | 145 | 234 | 261 | 188 | 162 |
| WA599 | 15 | 105 | 153 | 240 | 261 | 201 | 179 |
| WA917 | 15 | 105 | 155 | 240 | 217 | 195 | 168 |
| WA917 | 15 | 105 | 161 | 254 | 261 | 203 | 173 |
| FB1 16 | 105 | 137 | 234 | 214 | 191 | 168 |  |
| FB1 16 | 105 | 155 | 264 | 244 | 201 | 175 |  |
| FB1075 | 16 | 105 | 137 | 254 | 203 | 195 | 166 |
| FB1075 | 16 | 105 | 147 | 254 | 217 | 195 | 183 |
| FB1076 | 16 | 103 | 137 | 254 | 217 | 195 | 166 |
| FB1076 | 16 | 105 | 147 | 254 | 236 | 195 | 171 |
| FB1084 | 16 | 103 | 139 | 237 | 228 | 191 | 164 |
| FB1084 | 16 | 105 | 165 | 250 | 236 | 191 | 177 |
| FB1085 | 16 | 103 | 149 | 254 | 207 | 195 | 168 |
| FB1085 | 16 | 105 | 165 | 254 | 236 | 201 | 175 |
| FB11.3 | 16 | 105 | 137 | 230 | 254 | 195 | 168 |
| FB11.3 | 16 | 105 | 147 | 230 | 254 | 195 | 183 |
| FB17.25 | 16 | 105 | 137 | 234 | 228 | 188 | 166 |
| FB17.25 | 16 | 105 | 137 | 254 | 228 | 191 | 168 |
| FB2 16 | 99 | 137 | 234 | 224 | 191 | 166 |  |
| FB2 16 | 105 | 147 | 237 | 228 | 191 | 168 |  |
| FB21.3 | 16 | 99 | 155 | 234 | 269 | 195 | 173 |
| FB21.3 | 16 | 105 | 157 | 234 | 274 | 201 | 175 |
| FB27.25 | 16 | 103 | 137 | 234 | 228 | 188 | 158 |
| FB27.25 | 16 | 105 | 149 | 237 | 269 | 197 | 179 |
| FB3 16 | 105 | 133 | 234 | 203 | 195 | 171 |  |
| FB3 16 | 105 | 173 | 247 | 217 | 195 | 181 |  |

| FB6 16 | 105 | 137 | 221 | 232 | 191 | 164 |  |
| --- | --- | --- | --- | --- | --- | --- | --- |
| FB6 16 | 105 | 149 | 225 | 265 | 191 | 171 |  |
| FB7 16 | 105 | 149 | 237 | 224 | 201 | 164 |  |
| FB7 16 | 105 | 167 | 254 | 228 | 201 | 183 |  |
| FB8 16 | 99 | 141 | 234 | 228 | 191 | 168 |  |
| FB8 16 | 99 | 149 | 237 | 269 | 191 | 171 |  |
| FB90216 | 105 | 137 | 234 | 228 | 191 | 171 |  |
| FB90216 | 105 | 145 | 240 | 236 | 197 | 175 |  |
| FB90316 | 99 | 149 | 234 | 214 | 188 | 164 |  |
| FB90316 | 105 | 173 | 240 | 254 | 191 | 171 |  |
| FB90416 | 105 | 147 | 254 | 207 | 191 | 173 |  |
| FB90416 | 109 | 161 | 258 | 261 | 195 | 173 |  |
| FB90516 | 105 | 137 | 230 | 217 | 188 | 171 |  |
| FB90516 | 105 | 137 | 234 | 277 | 208 | 171 |  |
| FB90716 | 105 | 149 | 230 | 236 | 188 | 171 |  |
| FB90716 | 107 | 167 | 254 | 248 | 188 | 179 |  |
| FB90816 | 103 | 137 | 234 | 236 | 195 | 173 |  |
| FB90816 | 105 | 149 | 278 | 244 | 197 | 175 |  |
| FB90916 | 103 | 137 | 254 | 236 | 191 | 166 |  |
| FB90916 | 105 | 145 | 261 | 254 | 203 | 179 |  |
| FB91016 | 105 | 137 | 247 | 244 | 188 | 158 |  |
| FB91016 | 105 | 157 | 247 | 244 | 191 | 168 |  |
| FB91116 | 103 | 157 | 240 | 221 | 188 | 171 |  |
| FB91116 | 105 | 165 | 254 | 269 | 197 | 173 |  |
| FB91216 | 105 | 137 | 247 | 221 | 191 | 158 |  |
| FB91216 | 105 | 139 | 254 | 232 | 197 | 164 |  |
| FB91316 | 103 | 173 | 240 | 203 | 188 | 166 |  |
| FB91316 | 105 | 177 | 244 | 236 | 191 | 179 |  |
| FB91416 | 103 | 145 | 230 | 207 | 188 | 166 |  |
| FB91416 | 105 | 177 | 237 | 224 | 197 | 173 |  |
| PA1052 | 17 | 99 | 151 | 218 | 217 | 193 | 160 |
| PA1052 | 17 | 105 | 153 | 230 | 240 | 193 | 193 |
| PA1053 | 17 | 99 | 151 | 237 | 217 | 193 | 175 |
| PA1053 | 17 | 103 | 153 | 240 | 240 | 193 | 183 |
| PA1054 | 17 | 103 | 139 | 218 | 217 | 188 | 160 |

| PA818 | 17 | 103 | 143 | 230 | 248 | 188 | 168 |
| --- | --- | --- | --- | --- | --- | --- | --- |
| PA818 | 17 | 105 | 153 | 254 | 248 | 188 | 171 |
| PA830 | 17 | 105 | 145 | 230 | 236 | 188 | 168 |
| PA830 | 17 | 105 | 147 | 240 | 240 | 188 | 198 |
| PA831 | 17 | 105 | 139 | 240 | 244 | 193 | 160 |
| PA831 | 17 | 105 | 149 | 278 | 274 | 193 | 191 |
| PA832 | 17 | 105 | 147 | 237 | 240 | 195 | 166 |
| PA832 | 17 | 105 | 153 | 250 | 244 | 195 | 168 |
| PA833 | 17 | 99 | 143 | 237 | 248 | 188 | 177 |
| PA833 | 17 | 103 | 153 | 250 | 277 | 195 | 181 |
| PA834 | 17 | 103 | 137 | 237 | 261 | 195 | 179 |
| PA834 | 17 | 103 | 139 | 240 | 265 | 195 | 179 |
| PA835 | 17 | 99 | 143 | 234 | 228 | 191 | 168 |
| PA835 | 17 | 103 | 147 | 264 | 240 | 191 | 177 |
| PA836 | 17 | 103 | 137 | 237 | 217 | 188 | 168 |
| PA836 | 17 | 103 | 145 | 247 | 248 | 188 | 168 |
| PA881 | 17 | 105 | 143 | 244 | 236 | 193 | 168 |
| PA881 | 17 | 105 | 153 | 244 | 244 | 193 | 168 |
| PA882 | 17 | 105 | 151 | 223 | 269 | 201 | 168 |
| PA882 | 17 | 105 | 153 | 240 | 269 | 201 | 181 |
| PA883 | 17 | 99 | 153 | 223 | 211 | 188 | 168 |
| PA883 | 17 | 105 | 153 | 234 | 254 | 195 | 179 |
| PA884 | 17 | 99 | 149 | 223 | 207 | 197 | 166 |
| PA884 | 17 | 105 | 153 | 258 | 254 | 197 | 179 |
| PA885 | 17 | 105 | 145 | 240 | 217 | 188 | 173 |
| PA885 | 17 | 105 | 149 | 284 | 240 | 193 | 175 |
| PA886 | 17 | 105 | 139 | 237 | 228 | 193 | 179 |
| PA886 | 17 | 105 | 151 | 240 | 244 | 203 | 181 |
| PA887 | 17 | 103 | 147 | 237 | 224 | 193 | 181 |
| PA887 | 17 | 105 | 153 | 244 | 254 | 193 | 181 |
| PA888 | 17 | 99 | 143 | 247 | 240 | 191 | 179 |
| PA888 | 17 | 99 | 143 | 264 | 248 | 193 | 191 |
| PA889 | 17 | 105 | 143 | 230 | 254 | 193 | 160 |
| PA889 | 17 | 105 | 153 | 278 | 254 | 193 | 171 |
| PA890 | 17 | 99 | 149 | 218 | 217 | 188 | 164 |

| WE241 | 18 | 105 | 133 | 244 | 240 | 191 | 171 |
| --- | --- | --- | --- | --- | --- | --- | --- |
| WE241 | 18 | 105 | 167 | 247 | 277 | 197 | 171 |
| WE242 | 18 | 105 | 145 | 230 | 232 | 191 | 158 |
| WE242 | 18 | 105 | 147 | 234 | 244 | 191 | 168 |
| WE512 | 18 | 105 | 137 | 234 | 207 | 195 | 171 |
| WE512 | 18 | 105 | 147 | 247 | 274 | 195 | 171 |
| WE529 | 18 | 99 | 153 | 234 | 207 | 195 | 171 |
| WE529 | 18 | 107 | 153 | 250 | 236 | 208 | 185 |
| WE586 | 18 | 105 | 133 | 237 | 228 | 188 | 162 |
| WE586 | 18 | 105 | 153 | 244 | 240 | 201 | 171 |
| WE602 | 18 | 99 | 131 | 247 | 214 | 193 | 171 |
| WE602 | 18 | 105 | 167 | 254 | 240 | 201 | 198 |
| WE608 | 18 | 105 | 133 | 240 | 248 | 201 | 162 |
| WE608 | 18 | 105 | 135 | 244 | 248 | 203 | 193 |
| WE609 | 18 | 105 | 133 | 240 | 244 | 193 | 162 |
| WE609 | 18 | 105 | 135 | 244 | 248 | 193 | 181 |
| WE610 | 18 | 105 | 131 | 240 | 248 | 201 | 162 |
| WE610 | 18 | 105 | 135 | 244 | 248 | 203 | 181 |
| WE611 | 18 | 105 | 131 | 234 | 244 | 193 | 162 |
| WE611 | 18 | 107 | 135 | 240 | 261 | 201 | 171 |
| WE67 18 | 105 | 133 | 230 | 224 | 193 | 171 |  |
| WE67 18 | 105 | 155 | 234 | 228 | 193 | 175 |  |
| WE68 18 | 105 | 143 | 237 | 211 | 188 | 175 |  |
| WE68 18 | 105 | 165 | 240 | 254 | 193 | 175 |  |
| WE69 18 | 105 | 135 | 244 | 217 | 191 | 168 |  |
| WE69 18 | 105 | 137 | 247 | 254 | 197 | 171 |  |
| WE70 18 | 105 | 133 | 234 | 232 | 201 | 171 |  |
| WE70 18 | 109 | 155 | 254 | 261 | 208 | 171 |  |
| WE71 18 | 99 | 143 | 250 | 214 | 191 | 171 |  |
| WE71 18 | 103 | 155 | 264 | 214 | 191 | 171 |  |
| WE72 18 | 103 | 133 | 234 | 240 | 191 | 171 |  |
| WE72 18 | 105 | 173 | 237 | 254 | 208 | 175 |  |
| WE74 18 | 105 | 135 | 230 | 232 | 195 | 171 |  |
| WE74 18 | 105 | 143 | 230 | 236 | 201 | 193 |  |
| WE75 18 | 105 | 133 | 223 | 240 | 191 | 175 |  |
| WE75 18 | 107 | 133 | 237 | 265 | 201 | 185 |  |

| WE96 18 | 105 | 143 | 230 | 244 | 193 | 168 |
| --- | --- | --- | --- | --- | --- | --- |
| WE96 18 | 105 | 143 | 264 | 244 | 197 | 177 |
| WE97 18 | 105 | 133 | 240 | 217 | 195 | 171 |
| WE97 18 | 109 | 159 | 250 | 236 | 195 | 198 |
| WE98 18 | 105 | 135 | 230 | 240 | 191 | 171 |
| WE98 18 | 105 | 139 | 240 | 261 | 203 | 193 |
| MI1 19 | 103 | 153 | 234 | 200 | 191 | 166 |
| MI1 19 | 103 | 157 | 240 | 254 | 197 | 173 |
| MI13 19 | 99 | 153 | 230 | 236 | 191 | 173 |
| MI13 19 | 105 | 153 | 261 | 236 | 203 | 173 |
| MI2 19 | 99 | 133 | 230 | 236 | 191 | 164 |
| MI2 19 | 105 | 143 | 237 | 261 | 197 | 168 |
| MI207 19 | 99 | 149 | 221 | 203 | 193 | 168 |
| MI207 19 | 105 | 153 | 230 | 211 | 197 | 179 |
| MI26 19 | 103 | 133 | 254 | 248 | 188 | 158 |
| MI26 19 | 105 | 153 | 271 | 261 | 191 | 164 |
| MI3 19 | 99 | 151 | 240 | 232 | 188 | 160 |
| MI3 19 | 103 | 155 | 271 | 248 | 188 | 160 |
| MI44 19 | 103 | 133 | 230 | 217 | 191 | 162 |
| MI44 19 | 105 | 167 | 250 | 217 | 193 | 168 |
| MI49 19 | 105 | 149 | 244 | 248 | 188 | 168 |
| MI49 19 | 107 | 167 | 244 | 269 | 193 | 171 |
| MI51 19 | 103 | 133 | 254 | 248 | 188 | 158 |
| MI51 19 | 105 | 153 | 271 | 261 | 191 | 164 |
| MI548 19 | 99 | 155 | 230 | 203 | 197 | 164 |
| MI548 19 | 107 | 155 | 254 | 221 | 208 | 179 |
| MI549 19 | 99 | 147 | 237 | 203 | 197 | 177 |
| MI549 19 | 99 | 151 | 250 | 228 | 201 | 179 |
| MI550 19 | 105 | 149 | 240 | 224 | 193 | 166 |
| MI550 19 | 107 | 155 | 254 | 224 | 203 | 168 |
| MI555 19 | 105 | 133 | 247 | 244 | 193 | 168 |
| MI555 19 | 107 | 133 | 247 | 261 | 197 | 179 |
| MI58 19 | 99 | 143 | 223 | 232 | 193 | 171 |
| MI58 19 | 111 | 151 | 237 | 232 | 193 | 173 |
| MI59 19 | 103 | 167 | 234 | 224 | 188 | 160 |
| MI59 19 | 105 | 167 | 261 | 224 | 191 | 164 |
| MI60 19 | 103 | 147 | 225 | 221 | 188 | 151 |

| MI620 | 19 | 99 | 153 | 237 | 248 | 191 | 164 |
| --- | --- | --- | --- | --- | --- | --- | --- |
| MI620 | 19 | 103 | 157 | 247 | 277 | 193 | 173 |
| MI621 | 19 | 103 | 155 | 230 | 228 | 193 | 162 |
| MI621 | 19 | 107 | 165 | 234 | 240 | 201 | 166 |
| MI63 | 19 | 99 | 139 | 237 | 224 | 193 | 160 |
| MI63 | 19 | 103 | 155 | 237 | 261 | 193 | 160 |
| MI64 | 19 | 103 | 147 | 234 | 217 | 188 | 162 |
| MI64 | 19 | 105 | 155 | 240 | 244 | 193 | 166 |
| MI65 | 19 | 105 | 139 | 234 | 224 | 191 | 160 |
| MI65 | 19 | 107 | 181 | 234 | 240 | 191 | 162 |
| MI698 | 19 | 105 | 151 | 261 | 240 | 191 | 175 |
| MI698 | 19 | 107 | 161 | 261 | 240 | 191 | 175 |
| MI699 | 19 | 103 | 137 | 234 | 203 | 188 | 160 |
| MI699 | 19 | 105 | 137 | 250 | 207 | 191 | 193 |
| MI9 | 19 | 105 | 167 | 223 | 228 | 195 | 164 |
| MI9 | 19 | 105 | 181 | 230 | 254 | 203 | 168 |
